# Novel Bradykinin-Potentiating Peptides and Three-Finger Toxins from Viper Venom: Combined NGS Venom Gland Transcriptomics and Quantitative Venom Proteomics of the *Azemiops feae* Viper

**DOI:** 10.3390/biomedicines8080249

**Published:** 2020-07-28

**Authors:** Vladislav V. Babenko, Rustam H. Ziganshin, Christoph Weise, Igor Dyachenko, Elvira Shaykhutdinova, Arkady N. Murashev, Maxim Zhmak, Vladislav Starkov, Anh Ngoc Hoang, Victor Tsetlin, Yuri Utkin

**Affiliations:** 1 Federal Research and Clinical Centre of Physical-Chemical Medicine of Federal Medical Biological Agency, 119435 Moscow, Russia; daniorerio34@gmail.com; 2 Shemyakin-Ovchinnikov Institute of Bioorganic Chemistry RAS, 117997 Moscow, Russia; rustam.ziganshin@gmail.com (R.H.Z.); mzhmak@gmail.com (M.Z.); vladislavstarkov@mail.ru (V.S.); victortsetlin3f@gmail.com (V.T.); 3 Institute of Chemistry and Biochemistry, Freie Universität Berlin, 14195 Berlin, Germany; chris.weise@biochemie.fu-berlin.de; 4 Branch of the Shemyakin-Ovchinnikov Institute of Bioorganic Chemistry, Russian Academy of Sciences, Pushchino, 142290 Moscow Region, Russia; dyachenko@bibch.ru (I.D.); shaykhutdinova@bibch.ru (E.S.); murashev@bibch.ru (A.N.M.); 5 Institute of Applied Materials Science, Vietnam Academy of Science and Technology, Ho Chi Minh City 700000, Vietnam; hnanh52@yahoo.com

**Keywords:** Feae’s viper, *Azemiops feae*, venom, venom gland, proteomics, transcriptomic, bradykinin-potentiating peptides, three-finger toxins

## Abstract

Feae’s viper *Azemipos feae* belongs to the Azemiopinae subfamily of the Viperidae family. The effects of Viperidae venoms are mostly coagulopathic with limited neurotoxicity manifested by phospholipases A2. From *A. feae* venom, we have earlier isolated azemiopsin, a novel neurotoxin inhibiting the nicotinic acetylcholine receptor. To characterize other *A. feae* toxins, we applied label-free quantitative proteomics, which revealed 120 unique proteins, the most abundant being serine proteinases and phospholipases A2. In total, toxins representing 14 families were identified, among which bradykinin-potentiating peptides with unique amino acid sequences possessed biological activity in vivo. The proteomic analysis revealed also basal (commonly known as non-conventional) three-finger toxins belonging to the group of those possessing neurotoxic activity. This is the first indication of the presence of three-finger neurotoxins in viper venom. In parallel, the transcriptomic analysis of venom gland performed by Illumina next-generation sequencing further revealed 206 putative venom transcripts. Together, the study unveiled the venom proteome and venom gland transciptome of *A. feae*, which in general resemble those of other snakes from the Viperidae family. However, new toxins not found earlier in viper venom and including three-finger toxins and unusual bradykinin-potentiating peptides were discovered.

## 1. Introduction

Snake venoms are complex mixtures of peptides and proteins that are evolved in the process of evolution for protection from predators and for hunting. Two major action strategies can be traced in the venoms: a paralytic one inherent predominantly to elapids and colubrids and a coagulopathic one inherent mostly to viperids. To date, it has been established that, despite the huge variety of toxins, they can all be attributed to a limited number of families either possessing enzymatic activities (e.g., phospholipases A_2_ (PLA2), snake venom serine proteases (SVSP), snake venom metalloproteinases (SVMP)) or without such activities (e.g., lectins, three-finger toxins (3FTx), Kunitz-type inhibitors, natriuretic peptides). The variation in the composition of venoms occurs at all taxonomic levels [1], but the most drastic differences are evident at the level of families and subfamilies. As coagulopathies are induced mostly through an enzymatic pathway, in general, enzymes are the main components of viperid venoms. Paralytic effects, on the other hand, are produced more often by proteins without enzymatic activity (e.g., three-finger neurotoxins), albeit non-enzymatic proteins are not the main components in all elapid venoms. For example, in krait *Bungarus fasciatus* venom, PLA2 enzymes comprise about 70% of all venom proteins [2]. Although traditional methods for characterization of venoms based on chromatographic separation are still in use, the modern proteomic approaches yield more detailed and complete information. The proteomes of about two hundred snake species have been characterized so far [3,4], more than half of them (about 100 species) from viperids. Viperids (Viperidae family) are divided into two major subfamilies, the Viperinae (Old World or pitless vipers) and the Crotalinae (pit vipers). One more taxon, Azemiopinae, is recognized within the Viperidae; recent data confirm Azemiopinae as the sister group of the Crotalinae [5,6]. Nowadays, Azemiopinae include the genus Azemiops, the species composition of which is currently the subject of discussion. Recently, in addition to the well-established *Azemiops feae* species, a new one *Azemiops kharini* was included in the genus [7]. However, new data indicate that they are rather one species [8].

Data about venom composition for these fairly rare snakes are limited and available for *A. feae* only. Earlier investigation of the enzymatic activity of this venom showed that it had no blood clotting, hemorrhagic, or myolytic activities [9]. Recent studies confirmed the absence of coagulopathic effects for *A. feae* venom [10]. In another study, PLA2 and plasminogen activator homologs were found in the venom [11]. Three PLA2s were isolated from *A. feae* venom, and two of them contained Asn49 in their active center and were catalytically inactive [12]. cDNAs encoding cysteine-rich secretory protein (CRISP) [12], natriuretic-peptide precursors [13] and 3Ftx [14] were cloned from *A. feae*. However, there are no data about the presence of the corresponding proteins in *A. feae* venom. At the same time, natriuretic-peptide precursors contain sequences of a unique peptide neurotoxin, which was isolated by us from the venom and called azemiopsin [15]. Azemiopsin is a primitive neurotoxin; it consists of 21 residues and does not contain cysteine residues. By its capacity to block the nicotinic acetylcholine receptor, azemiopsin resembles waglerin, a disulfide-containing peptide from the *Tropedolaemus wagleri* venom, shares with it a homologous C-terminal hexapeptide and may be considered as a waglerin ancestor. These peptide neurotoxins are not typical for Viperidae venom. To find out what the other components are, we have analyzed *A. feae* venom in more depth using high-throughput proteomics. In parallel, the transcriptomic analysis of venom gland was performed by Illumina next-generation sequencing (NGS). About 70 toxins representing 14 toxins families were identified; among them, new toxins not found earlier in viper venom and including three-finger toxins and unusual bradykinin-potentiating peptides were discovered.

## 2. Experimental Section

### 2.1. Specimen Collection and Tissue Preparation

Specimen of *A. feae* was collected in Vinh Phuc Province, Vietnam. Venom gland was dissected 3 days after stimulation by milking and treated with RNAlater (Ambion, Thermo Fisher Scientific Inc, Waltham, MA, USA) as described by the vendor.

### 2.2. cDNA Library Preparation and Sequencing

For RNA extraction, venom gland dissected from one specimen of *A. feae* viper and stored in RNAlater (Ambion, Thermo Fisher Scientific Inc, Waltham, MA, USA) was used. Total RNA was isolated from the tissue sample by TRIzol kit (Invitrogen, Thermo Fisher Scientific Inc, Waltham, MA, USA), and its quality was assessed on an Agilent 2100 Bioanalyzer (Agilent Technologies, Santa Clara, CA, USA).

#### 2.2.1. cDNA Synthesis and cDNA Amplification

For ds cDNA synthesis, the SMART approach [16] was applied. SMART-prepared cDNA was amplified by PCR. Primer annealing mixture (5 µL) containing 0.3 μg of total RNA; 10 pmol of SMART Oligo II oligonucleotide (5′-AAGCAGTGGTATCAACGCAGAGTACGCrGrGrG-3′) and 10 pmol of CDS-T22 primer (5′-AAGCAGTGGTATCAACGCAGAGTTTTTGTTTTTTTCTTTTTTTTTTVN-3′) was prepared, incubated for 2 min at 72 °C and was kept for 2 min on ice. The annealed primer-RNA was mixed with Reverse Transcriptase in a final volume of 10 μL, containing 1X First-Strand Buffer (50 mM Tris-HCl (pH 8.3); 75 mM KCl; 6 mM MgCl_2_), 2 mM DTT and 1 mM of each dNTP to start the first-strand cDNA synthesis. This reaction mixture was maintained at 42 °C for 2 h in a thermostat and then cooled on ice. After dilution by 5 times with TE buffer, the first-strand cDNA was incubated for 7 min at 70 °C and then was amplified by Long-Distance PCR (Barnes, 1994). PCR reaction mixture (50 μL) contained 1 μL diluted first-strand cDNA, 1 μL 50× Advantage reaction buffer (Clontech, Takara Bio, Mountain View, CA, USA), 200 μM dNTPs; 0.3 μM SMART PCR primer (5′-AAGCAGTGGTATCAACGCAGAGT-3′) and 1 μL 50× Advantage Polymerize mix (Clontech, Takara Bio, Mountain View, CA, USA). PCR was performed on MJ Research PTC-200 DNA Thermal Cycler under the following program: 95 °C −7 s, 65 °C −20 s, 72 °C −3 min, 17 cycles. Amplified cDNA PCR product was purified using QIAquick PCR Purification Kit (Qiagen, Venlo, Netherlands).

#### 2.2.2. Illumina Sequencing

The standard Illumina protocol was applied to prepare libraries for DNA using TruSeq Illumina DNA sample preparation kit. NGS was performed on Illumina Genome Analyzer IIx in the paired-end sequencing mode (2 × 72 bp).

### 2.3. NGS Data Analysis

Sequenced data from Illumina GAIIx were transformed by base calling into sequence data, called the raw data or raw reads, and were stored in FASTQ format. For quality control checks on raw sequence data, we used FastQC (version 0.11.5). Trimmomatic (version 0.38) was applied to trim the adapter sequences used for cDNA synthesis. For de novo transcriptome assembly, we used Trinity software (version r20131110) with the default settings. Contigs from Trinity were translated using all six reading frames to obtain “Predicted protein SET 1”. Local BLAST (BlastX threshold value of e = 1 × 10^−6^, matrix BLOSUM-62) and the non-redundant database were used for the grouping and annotation of contigs. Blast2GO (version 5.2.1) was used for Taxonomy, Gene Ontology (GO) and EuKaryotic Orthologous Groups (KOG) analysis for functional annotation of contigs. TransDecoder was applied to identify putative ORFs with a minimum length of 100 amino acids. The ORF database (10997 sequences) generated with Transdecoder called “Predicted protein SET 2” was used for proteomics.

### 2.4. Other Computational Tool

Translation of transcriptome sequences in all 6 frames to amino acid sequences was done with Nucleotide Sequence Translation EMBOSS Transeq/EMBOSS Sixpack. The ORF database (120510 sequences) generated with EMBOSS Sixpack was used for proteomics. Prinseq lite v.0.20.4 was used for reads quality and length trimming. ClustalW2 and MUSCLE algorithms integrated into package Jalview v.2.11.1.0 were used to perform multiple alignment construction and visualization [17]. For calculating expression levels (Transcripts Per Kilobase Million (TPM) and Reads Per Kilobase Million (RPKM), we used CLC Genomic Workbench 10 (Qiagen, Venlo, Netherlands) with the default settings.

### 2.5. BioProject and Raw Sequence Data

Sequence data from the venom gland transcriptome of the *A. feae* have been deposited in National Centre for Biotechnology Information (NCBI) Sequence Read Archive with the accession number SRR8177476 under Bioproject PRJNA504599.

### 2.6. Reduction, Alkylation and Digestion of the Proteins

The proteins were reduced, alkylated and digested as described previously [18] with small variations. In brief, to a venom sample (10 μg), sodium deoxycholate (SDC) buffer (pH 8.5) for reduction and alkylation was added so as to achieve the final concentration of proteins, Tris, SDC, tris(2-carboxyethyl)phosphine and 2-chloroacetamide of 0.5 mg/mL, 100 mM, 1% (*w*/*v*), 10 mM and 20 mM, respectively. The reaction mixture was incubated at 95 °C for 10 min and cooled to ambient temperature. Digestion with trypsin (solution in equal volume of 100 mM Tris, pH 8.5) at a 1:100 (*w*/*w*) ratio was performed at 37 °C overnight.

### 2.7. Tryptic Peptides Desalting

Peptides were desalted with application of SDB-RPS StageTips, which were made as previously described [19]. In brief, two small portions of the 3M Empore SDB-RPS membrane were cut with a blunt-tipped Hamilton needle (part no. 91014: gauge 14, type Point 3, metal (N) hub) and pressed into the 200-µL pipette tip using 1/16 PEEK tubing (1535, Upchurch Scientific, Thermo Fisher Scientific Inc, Waltham, MA, USA). As a StageTip holder, 2 mL microcentrifuge tube with a hole punched in the cap (O-tube) was used. O-tube and SDB-RPS StageTip composed a Spin block. An equal volume of 2% (*v*/*v*) TFA was added to the tryptic peptide solution obtained after overnight hydrolysis, and the solution thus obtained was applied to StageTip using centrifugation at 200 g. StageTip was cleaned by washing first with mixture of 50 µL ethylacetate with 50 µL 1% (*v*/*v*) TFA (3 times), then 50 µL 1% (*v*/*v*) TFA and finally 50 µL 0.2% (*v*/*v*) TFA. The elution of peptides was achieved by application of 60 μL 50% (*v*/*v*) acetonitrile containing 5% (*v*/*v*) NH_4_OH; the eluted peptides were freeze-dried and preserved at −80 °C. For further analysis, peptides were dissolved in 20 µL of 2% (*v*/*v*) acetonitrile containing 0.1% (*v*/*v*) TFA and treated with ultrasound for 2 min.

### 2.8. Liquid Chromatography and Mass Spectrometry

The column (25 cm with inner diameter of 75 μm) in-house packed with Aeris Peptide XB-C18 2.6 μm resin (Phenomenex, Torrance, CA, USA) was applied for the separation of peptides. An Ultimate 3000 Nano LC System (Thermo Fisher Scientific Inc, Waltham, MA, USA) was used for reverse-phase chromatography. The LC System was connected through a nanoelectrospray source (Thermo Fisher Scientific) to a Q Exactive HF mass spectrometer (Thermo Fisher Scientific). A linear 120-min gradient of 4–55% solvent B (80% (*v*/*v*) acetonitrile containing 0.1% (*v*/*v*) formic acid was used for the elution of peptides at a flow rate of 350 nL/min (40 °C). TopN method with an automatic switch between a full scan and up to 15 data-dependent MS/MS scans was applied for MS data acquisition. The target value of 3 × 10^6^ in the 300–1200 *m*/*z* range with a resolution of 60,000 and a maximum injection time of 60 ms was used for the full-scan MS spectra. A 1.4 *m*/*z* window and a fixed first mass of 100.0 *m*/*z* was used to perform isolation of precursors. A higher-energy dissociation (HCD) and a normalized collision energy of 28 eV were applied for fragmentation of precursors. An ion target value of 1 × 10^5^ in the 200–2000 *m*/*z* range with a resolution of 15,000 at *m*/*z* 400 and a maximum injection time of 30 ms were used for the acquisition of MS/MS scans. Six technical replicates were performed to analyze the sample by LC–MS/MS.

### 2.9. Data Analysis

Six technical replicates were used for MS measurements of venom sample. MS/MS-based qualitative proteome analysis of venom proteins was made using PEAKS Studio 8.0 build 20160908 software [20]. Peptide lists generated by the PEAKS Studio were searched against the “SET 2” FASTA database. Carbamidomethylation was set as a fixed modification for cysteine and acetylation for N-terminus, oxidations for methionine and deamidations for asparagine were set as variable modifications and specificity of protease was set to semitryptic. For peptide-spectrum matches, the false discovery rate (FDR) was fixed to 0.01 and the search of a reverse database was used to determine FDR. An allowed fragment mass deviation of 0.05 Da and an allowed initial precursor mass deviation up to 10 ppm were used to perform the peptide identification. If at least 1 unique peptide was found for a protein, it was regarded as identified reliably. MaxQuant program version 1.5.6.5 [21] was applied for label-free protein quantification. Carbamidomethylation was set as a fixed modification for cysteine and acetylation for N-terminus, oxidations for methionine and deamidations for asparagine were set as variable modifications and specificity of protease was set to semitryptic. The Andromeda search engine [22] was used for the search of peak lists against the “SET 1” FASTA database and a common contaminant database implemented in the search engine. The FDR of 0.01 was fixed for both proteins and peptides, and a minimum length of seven amino acids was used. An allowed fragment mass deviation of 20 ppm and an allowed initial precursor mass deviation up to 20 ppm were used to perform the peptide identification. Perseus (versions 1.5.5.1) [23] was applied to perform the downstream bioinformatics analysis. From the analyses were excluded groups of proteins which were identified only by site, from peptides identified also only in the reverse database oring belonged to the database of common contaminant. A minimum ratio count of 1 was applied to perform the label-free quantification. The iBAQ algorithm, implemented into the MaxQuant program [21], was used to quantify proteins in the venom sample. To generate a relative iBAQ (riBAQ) value representing the mole fraction of each protein [24], normalization of each protein’s iBAQ value against the sum of all iBAQ values was performed.

### 2.10. Characterization of Bradykinin-Potentiating Peptides by MALDI Mass Spectrometry

De novo peptide sequencing was performed by matrix-assisted laser desorption ionization-time of flight mass spectrometry (MALDI-TOF-MS/MS) using an Ultraflex-II TOF/TOF instrument (Bruker Daltonics, Bremen, Germany) equipped with a 200 Hz solid-state Smart beam™ laser.

HPLC-purified samples were applied using α-cyano-4-hydroxycinnamic acid (CHCA, saturated solution in 33% acetonitrile/0.1% trifluoroacetic acid) onto a polished-steel target. The instrument was operated in positive reflector mode and MS/MS spectra generated by spontaneous post-source decay were recorded using the LIFT cell technology [25]. The fragment spectra were interpreted and labeled manually.

### 2.11. Peptide Synthesis

The bradykinin-potentiating peptides (BPP) were prepared by peptide synthesis and purified by reverse-phase chromatography as described [26].

### 2.12. Blood Pressure Measurements

The experiments were carried out on adult male Sprague-Dawley (SD) rats (300–350 g of body weight). The World Health Organization’s International Guiding Principles for Biomedical Research Involving Animals were followed during experiments on animals. The experiments were approved by the Institutional Animal Care and Use Committee (IACUC No.536/18). Four groups of 6 rats in each were used for the study. Before the experiment, catheters were implanted into the jugular vein and carotid artery of rats under anesthesia (Zoletil-100 at a dose of 20 mg/kg and Rometar at a dose of 12 mg/kg). The arterial catheter was used to record blood pressure (mean BP) and the venous catheter for injection of test drugs (Y-BPP or R-BPP). Blood pressure parameters were recorded on conscious animals a day after surgery (to allow for recovery of the animals from anesthesia) continuously using a strain gauge pressure transducer linked to Powerlab ML125 system (AD Instrument, Australia). After 10 min of recording of baseline parameters, bradykinin was introduced (1st injection, 7 µg/kg); 10 min after bradykinin, for the experimental group, BPP (Y-BPP or R-BPP in two doses: 300 µg/kg or 1 mg/kg) was introduced (2nd injection). Then, on the 5th minute after BPP injection, bradykinin (7 µg/kg, 3rd introduction) was again introduced; on the 50th minute after beginning of the experiment, bradykinin was again given at the same dose (4th introduction). The last introduction of bradykinin was at the 80th minute (5th introduction). Data were then analyzed and mean BP ± SEM calculated. All quantitative data are presented as mean ± SD. Statistical analysis was performed by software Statistica for Window v.7.1 to compare the treated groups. Significance level was determined at *p* ≤ 0.05. No rats or data points were excluded from the reported analyses.

## 3. Results

### 3.1. Research Outline: RNA Sequencing and Analysis of the Initial Data

**Figure 1:**
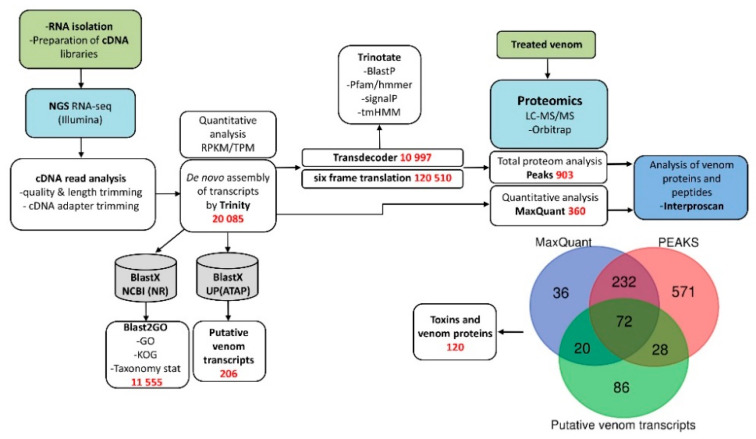
Analysis pipeline of the research implemented for *Azemiops feae*. GO—gene ontology, KOG—euKaryote Orthologous Groups, ATAP—Animal Toxin Annotation Project (UniProt).

To perform a comprehensive analysis of Feae’s viper *A. feae* venom, we used two complementary approaches (Figure 1). To study proteins and peptides present in the venom, quantitative proteomics was applied (Table S1), while the transcriptome of the venom gland was analyzed by NGS.

From the venom glands of *A. feae*, we generated 29,055,985 pairs of 75 bp reads that passed the Illumina filter (Table S2). Basic filters recommended for qualitative analysis of Illumina were applied to the raw reads, and the adapters used for cDNA synthesis were trimmed. Final cDNA short reads dataset was used for the de novo assembly of a transcriptome using the Trinity assembler. Trinity created 20,085 contigs (reconstructed transcripts) with (N50 = 781), connected to form 18,296 “genes” in total assembly statistics (Table S3).

Next, the gene ontology (GO), eukaryotic orthologous groups (KOG) and taxonomic affiliation of the contigs were analyzed using the Blast2GO program and the BlastX algorithm. The non-redundant database served as a reference database (Figures S1–S3). Furthermore, for identifying potential toxins in the transcriptome, local BlastX was used. As a reference database, we used all toxin sequences from the UniProt animal toxin annotation project (https://www.uniprot.org/program/Toxins). As a result, 206 putative venom transcripts clustered into 112 groups were identified (Table S4).

Finally, all contigs from Trinity were translated using all six reading frames in “Predicted protein SET 1” for subsequent validation by proteomics. The prediction of coding regions (or open read frames, ORFs) and annotation of transcriptomic data were carried out using Transdecoder and Trinotate. In addition, ORFs from Transdecoder were translated in “Predicted protein SET 2” for subsequent validation by proteomics. Both data sets were used to analyze the total proteome using the PEAKS Studio software. Transcriptome database was used for the quantitative analysis search in MaxQuant. As a result, 120 putative venom transcripts were confirmed using proteomics. A combination of these approaches allowed identifying proteins belonging to 43 protein families, among which 15 can be assigned to snake toxins (Figure 2) (Figures S4–S28).

### 3.2. Venom-Gland Transcriptome

**Figure 2:**
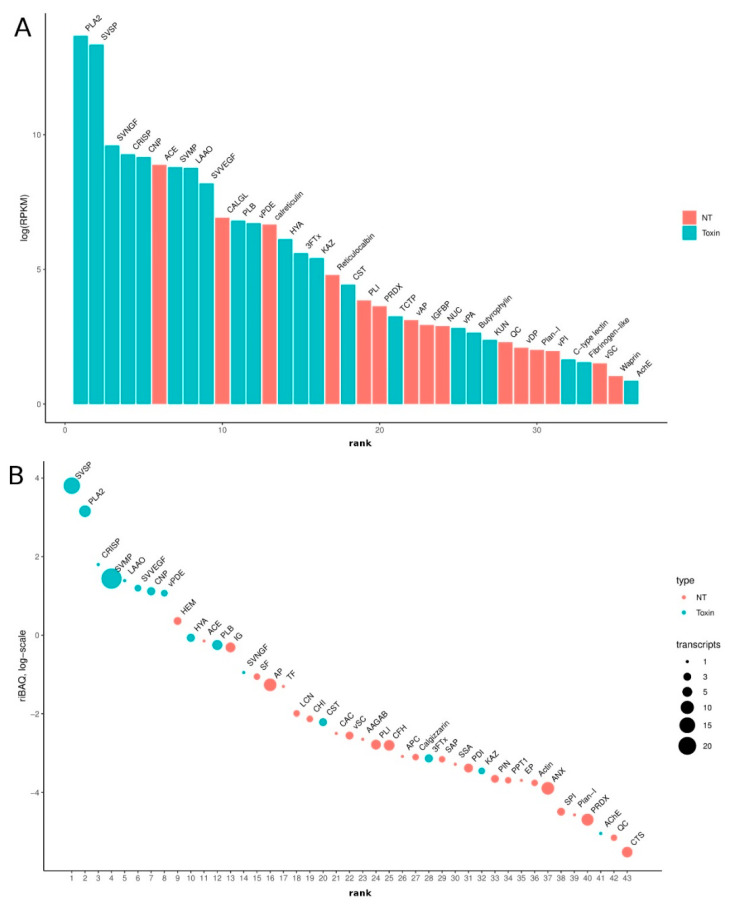
(**A**) Bar chart showing the distribution of Reads Per Kilobase Million (RPKM) values for all putative venom proteins (PVP) families found in transcriptome; (**B**) Scatter plot showing the distribution of riBAQ values of top 43 protein families. The diameter of the circle is proportional to the number of identified transcripts by quantitative proteomics. Abbreviations: 3FTx—Three-finger toxin, AAGAB—Alpha/Gamma adaptin binding protein, ACE—Angiotensinogenase, AChE—Acetylcholinesterase, ANX—Annexin, AP—Aminopeptidase, APC—Serum amyloid P-component, Butyrophylin—Butyrophylin-like, CAC—Calcyclin, CFH—Complement factor H, CHI—Chitinase, CNP—C-type natriuretic peptide (Azemiopsin included), CRISP—cysteine-rich secretory protein, CST—Cystatin, CTS—Cathepsin, EP—Endopeptidase, HEM—Hemoglobin, HYA—Hyaluronidase, IG—Immunoglobulin, IGFBP—Insulin-like growth factor-binding, KAZ—kazal serine protease inhibitor, KUN—Kunitz-type serine protease inhibitor, LAAO—L-amino acid oxidase, LCN—Lipocalin, NUC—5′-nucleotidase, PDI—Disulfide-isomerase, PLA2—phospholipases A2, PLB—Phospholipase B, Plan-I—Plancitoxin-1 (Deoxyribonuclease-2-alpha), PIN—Peptidyl-prolyl cis-trans isomerase, PLI—Phospholipase A2 inhibitor, PPT1—Palmitoyl- thioesterase 1, PRDX—Peroxiredoxin, QC—Glutaminyl-peptide cyclotransferase, SAP—Saposin-A, SF—Siderophilin, SPI—Serpin, SSA—Small serum 2, SVMP—Snake venom metalloproteinase, SVNGF—Snake-venom nerve growth factor, SVSP—Snake-venom serine proteinases, svVEGF—Vascular endothelial growth factor, TCTP—Translationally controlled tumor homolog, TF—Transferrin, vAP—Venom acid phoshatase, vDP—Venom dipeptidyl peptidase 4, vPA—Venom prothrombin activator, vPDE—Venom phosphodiesterase 1, vPI—Venom peptide isomerase, vSC—Serine carboxypeptidase, Waprin—Waprin-like.

We estimated the expression level of each transcript by mapping reads against a de novo transcriptome assembly. As expected, the level of representation of putative venom transcripts (toxins) is significantly higher than other classes of molecules (Figure 2A). Approximately 63% of total transcription was accounted for by the coding sequences of putative toxins, and the 54 most abundant transcripts in the transcriptome encoded putative toxins (Figure 2B).

**Figure 3:**
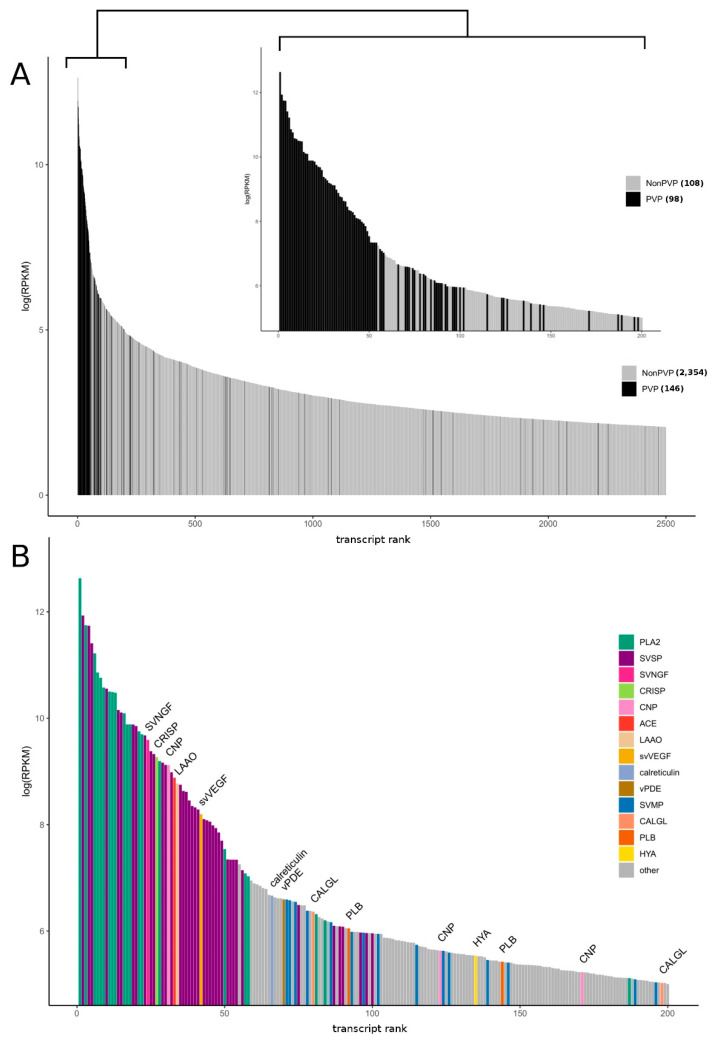
The overall expression patterns for the most abundant transcripts in *A. feae* venom gland tissue. (**A**) Expression of toxins and venom proteins (PVP—putative venom transcripts) dominates relative to other transcripts; (**B**) The venom-gland transcriptome of *A. feae* showed high expression and diversity of snake venom phospholipases A2 and serine proteinases. Expression levels of individual PVP transcripts are shown with toxin classes coded by color. Abbreviations: ACE—Angiotensinogenase, CALGL—Calmodulin, CNP—C-type natriuretic peptide (Azemiopsin included), CRISP—Cysteine-rich secretory protein, HYA—Hyaluronidase, LAAO—L-amino acid oxidase, PLA2—Phospholipases A2, PLB—Phospholipase B, SVMP—Snake venom metalloproteinase, SVNGF—Snake-venom nerve growth factor, SVSP—snake-venom serine proteinases, svVEGF—Vascular endothelial growth factor, vPDE—Venom phosphodiesterase 1.

We grouped all the toxin transcripts into main classes (families) (Figure 3). At the transcriptome level, the most abundant classes of toxin transcripts are PLA2 and SVSP, followed by the second group including snake venom nerve growth factor (svNGF), CRISP, C-type natriuretic peptide (CNP, including azemiopsin), angiotensinogenase (ACE), SVMP, L-amino acid oxidase (LAAO) and snake venom vascular endothelial growth factor (svVEGF). Such classes of proteins as PLB, venom phosphodiesterase (vPDE), HYA and 3FTx are significantly less abundant. Thus, the expression pattern of the *A. feae* venom gland is typical of viper snakes.

However, we detected a number of additional low-abundance toxins in the venom-gland transcriptome including 3FTxs and acetylcholinesterase not typical for vipers.

### 3.3. Venom Proteomics/Peptidomics

In this work, both venom proteome and peptidome for Feae’s viper *A. feae* were analyzed (Tables S1 and S5). Venom proteome was analyzed by LC–MS/MS after in-solution trypsin proteolysis. In our study, mass spectrometry analysis of *A. feae* venom revealed more than one hundred different proteins, some of them being identified earlier. Thus, PLA2 [11,12] and a plasminogen activator [10] were isolated from the venom, while the amino acid sequences of CRISP [12], several SVSPs (GenBank: ART88741.1, ART88740.1, ART88739.1, ART88738.1), three-finger toxin 3FTx-Aze-1 [14], SVNGF [27], and CNP precursors 1 and 2 [13] were deduced from cloned cDNA sequences. The peptide neurotoxin azemiopsin was isolated earlier from the *A. feae* venom as well [15].

Proteomic analysis in addition to the above-mentioned proteins, including those for which amino acid sequences were deduced from the cDNA, revealed in the venom the presence of LAAO, SVMP, venom epidermal growth factor, HYA, angiotensinogenase (renin-like aspartic protease), cystatin and ovomucoid (Kazal-type inhibitor-like protein).

The label-free quantification of venom proteins revealed that the most abundant venom proteins are SVSPs accounting for 44.8% (mole fraction) of total proteins. They are followed by PLA2s (25.8%), CRISP (6.0%), LAAO (4.0%), SVMP (3.9%), SVVEGF (3.3%), azemiopsin (bradykinin-potentiating and C-type natriuretic peptides, 3.0%) and PLB (2.9%). The contents of other venom proteins was less than one percent: HYA—0.9%, SVNGF—0.4%, cystatin (CST)—0.1%, 3FTx—0.04% and Kazal-type serine protease inhibitor (Kazal)—0.03%. The toxins present in *A. feae* venom at the level higher than 1% are considered in more detail.

#### 3.3.1. Serine Proteases

Using proteomics in *A. feae* venom, we identified 24 sequences (seven clusters) corresponding to snake venom serine proteases (SVSPs) (Table S4 and Figure S4). However, the assembled transcripts are incomplete and are truncated at either the 5′ or 3′ ends. This drawback prevents an adequate analysis for the classification of SVSPs to certain types. Nevertheless, when considering the alignment, it is seen that the closest homologs are the previously described proteases from *A. feae*, which are secreted trypsin-type proteases (ART88741.1, ART88740.1, ART88739.1, ART88738.1). The identified sequences include also those homologous to plasminogen activators, one of which was found in this venom earlier [11], thrombin-like enzymes and homologs of beta-fibrinogenase. All SVSPs from *A. feae* venom are homologous to those of other snakes from the Viperidae family. Enzymes homologous to plasminogen activators and thrombin-like are the most abundant in *A. feae* venom among SVSPs.

#### 3.3.2. Phospholipases A2

Five clusters PLA2s were found in *A. feae* venom and 11 sequences we identified by proteomics (Table S4 and Figure S5A). They are both acidic and basic enzymes from group II subfamily. The most abundant PLA2s (from about 1 to about 9% of total venom proteins) belong to the enzymatically active D49 sub-family. The minor PLA2s represent the N49 sub-subfamily (from 0.003 to 2%). We found all PLA2 subfamilies of N49a, with the exception of N49b previously discovered [12] in *A. feae* venom from Zhejiang province (China). In the found sequences, slight differences with reference ones are observed, which can be explained by genetic polymorphisms. The remaining sequences are intermediate isoforms of the previously described phospholipases of *A. feae* venom. New isoforms of basic PLA2s were also found. The protein sequence TR6913_c96_g4_i4 resembles basic PLA2s, and sequence TR6913_c96_g4_i6 has homology to the sequence of *Crotalus atrox* (QBA85153.1) (Figure S5A,B).

#### 3.3.3. Cysteine-Rich Seceretory Proteins

**Figure 4:**
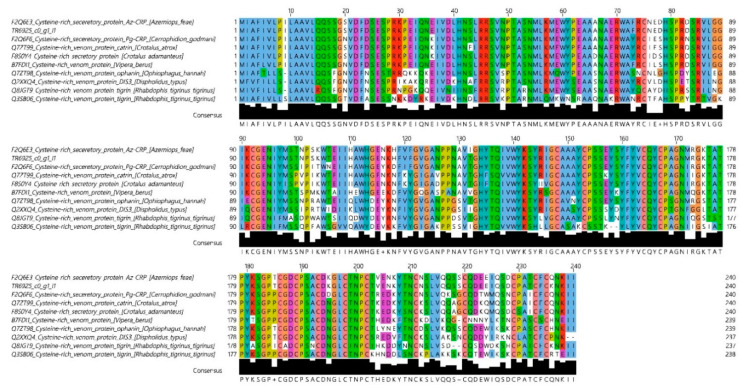
Amino acid sequence of *A. feae* CRISPs aligned with sequences of homologous proteins. TR6925_c0_g1_i2 is the sequence determined in this work. Alignment was generated by MUSCLE (Here and in all following captions MUSCLE is MUltiple Sequence Comparison by Log-Expectation) with ClustalX Colour Scheme: hydrophobic amino acid residues—blue; positively charged—red; negatively charged—magenta; polar—green; cysteines—pink; glycines—orange; prolines—yellow; aromatic—cyan; unconserved -white.

Only one CRISP was found in the *A. feae* venom (Figure 4). The CRISP amino acid sequence determined in this work is very similar to that of Az-CRP determined earlier for *A. feae* CRISP [12]. The two sequences differ only in two positions, 132 and 236 (Figure 4); Ile and Gln in Az-CRP are replaced by Thr and Lys, respectively, in the sequence determined in this work.

#### 3.3.4. L-Amino Acid Oxidase

LAAO is found in venoms of snakes from different families. Similarly to CRISP, this toxin is represented by only one protein in *A. feae* venom. *A. feae* LAAO has the highest similarity to that of the Okinawa pitviper (or Hime habu), *Ovophis okinavensis* from the Crotalinae subfamily (Figure S6).

#### 3.3.5. Snake Venom Metalloproteinase

In venoms of many species from the Viperidae family, SVMPs are the main components. For example, in *Bothrops atrox* venom, SVMPs represent more than half of total venom protein [28]. In *A. feae* venom, SVMPs account for only 3.9% of total protein and are represented by 16 enzymes (Figure S7). As in the case of SVSPs, only 5′ or 3′ terminal fragments were assembled, so no full-length protein sequences were obtained. Analysis of the domain organization (Figure S7B) allowed us to conclude that *A. feae* venom contains several isoforms of classical snake venom type III SVMP (Figure S7).

#### 3.3.6. Snake Venom Vascular Endothelial Growth Factor

In *A. feae* venom, svVEGF is represented by two proteins which account for 3.3% of total venom protein (Figure S8).

#### 3.3.7. Three-Finger Toxins

**Figure 5:**
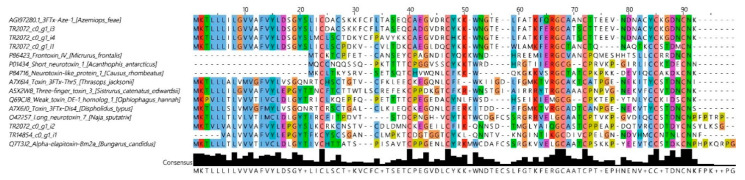
Three-finger toxins (3FTx). Comparison of amino sequences of *A. feae* toxins with those of known 3FTxs. TR2072 c0 g1 i3 is named 3FTx-Aze-2; TR2072 c0 g1 i4—3FTx-Aze-3; TR2072 c0 g1 i2—3FTx-Aze-4; TR14854 c0 g1 i1—3FTx-Aze-5; TR2072 c0 g1 i1—3FTx-Aze-6; TR13523 c0 g1 i1—3FTx-aze-7. Alignment was generated by MUSCLE with ClustalX Colour Scheme: hydrophobic amino acid residues—blue; positively charged—red; negatively charged—magenta; polar—green; cysteines—pink; glycines—orange; prolines—yellow; aromatic—cyan; unconserved—white.

Among the toxins present in *A. feae* venom at levels less than 1%, 3FTxs are of special interest. 3FTxs are the main components of venoms from Elapidae snakes. Transcripts of 3FTxs were identified in transcriptomes of several vipers. However, the neurotoxins were not found in viper venoms so far. Here, for the first time, we have found the 3FTxs in the venom of a snake from the Viperidae family. Even though the content of 3FTxs in *A. feae* venom is as low as 0.04%, there is no doubt about the presence of these toxins. Five three-finger toxins were found in *A. feae* venom (Figure 5); according to their amino acid sequences, they represent so-called basal (commonly known as non-conventional) toxins containing the fifth disulfide bond in the first loop [29,30]. The recombinant toxin 3FTx-Aze-2 showed the capacity to interact with muscle type and neuronal nicotinic acetylcholine receptors [31].

#### 3.3.8. Bradykinin-Potentiating Peptides and Azemiopsin

**Figure 6:**
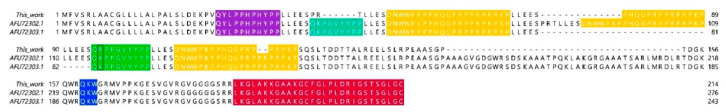
Amino acid sequences of C-type natriuretic precursors deduced from DNA sequences. Azemiopsin—yellow, Y-BPP—lilac, R-BPP and its Lys-analog—light green, putative shortened R-BPP analog—turquoise, putative tripeptide metalloproteinase inhibitor—blue, atrial natriuretic peptide—red.

Azemiopsin is a unique peptide toxin isolated earlier by us from *A. feae* venom [14]. According to the present analysis, its content in the venom is equal to 3.0%. The amino acid sequence of azemiopsin was identified previously within C-type natriuretic precursors [13] (Figure 6). It is interesting to note that several azemiopsin sequences are present within precursor sequences. Moreover, elongated analogs of azemiopsin (M = 2540) with additional N-terminal residues—SDNWWPKPPHQGPRPPRPRPKP (M = 2627) and ESDNWWPKPPHQGPRPPRPRPKP (M = 2756)—were identified in the venom (Table S5). When these peptides were fragmented in the mass spectrometer, the C-terminal ions remained constant, while for the N-terminal b-ions, corresponding mass shifts were observed (Figure S29).

**Figure 7:**
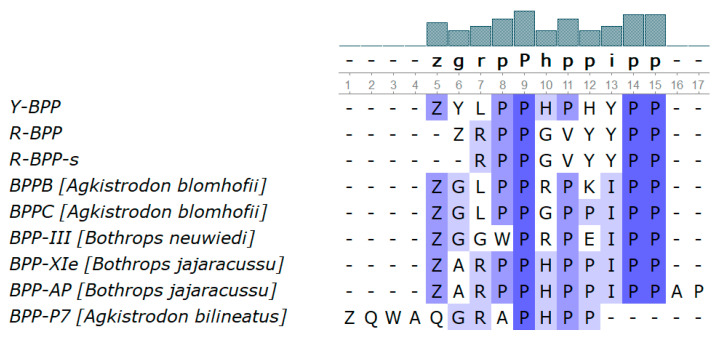
Bradykinin-potentiating peptides (BPP). Comparison of amino acid sequences of *A. feae* peptides with those of known bradykinin-potentiating peptides (pyroGlu = Z). The number of each consensus letter is represented by a histogram on the top of the figure. Alignment was generated by MUSCLE with percentage identity coloring scheme where the white color is corresponding to 0% identity and the darkest color to 100%.

During isolation of azemiopsin, we found several peptides with molecular masses of 1328.64 (Y-BPP), 1156.57 (R-BPP) and 1045.5 Da (R-BPP-s). The amino acid sequences of these peptides were established by de novo MALDI MS/MS sequencing (Figure S30). The determined sequences are shown in Figure 6. The peptides Y-BPP and R-BPP contain pyroglutamic acid at their N-terminus; the amino acid sequence of R-BPP-s corresponds to an N-terminally truncated form of R-BPP. The determined sequences show homology to some bradykinin-potentiating peptides (Figure 7). However, the sequences between the two PP repeats are unique for *A. feae* peptides. It should be mentioned that the tripeptide sequence QKW is present in the CNP precursor (Figure 6). This tripeptide may represent an endogenous metalloproteinase inhibitor ZKW (Z = pyroGlu) found in venoms of several Viperidae species. The C-type natriuretic precursors contain the sequence of C-type natriuretic peptide (Figure 6). This peptide was also found in the peptidome of *A. feae* venom (Table S5).

#### 3.3.9. Biological Activity of New Bradykinin-Potentiating Peptides

**Figure 8:**
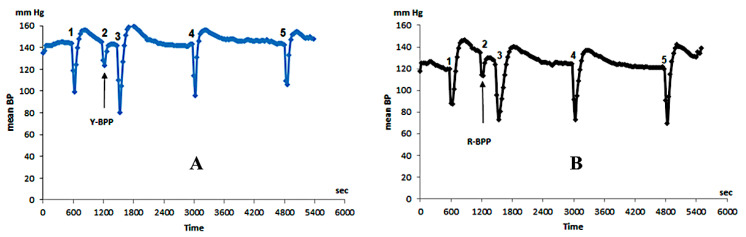
Bradykinin-potentiating effects of *A. feae* peptides Y-BPP and R-BPP. Changes in mean blood pressure (BPs) (absolute values) after administration of bradykinin at a dose of 7 μg/kg (injections at points 1, 3, 4, 5) and Y-BPP (**A**) or R-BPP (**B**) at a dose of 300 μg/kg (injection at point 2).

**Figure 9:**
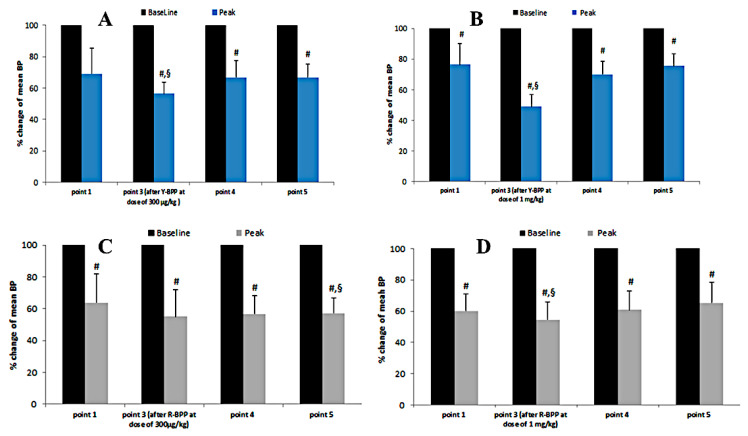
Percent changes from baseline for mean BPs after injections of bradykinin at a dose of 7 μg/kg in response to bradykinin-potentiating effects of *A. feae* peptides Y-BPP (**A**,**B**) and R-BPP (**C**,**D**) at two doses. # *p* ≤ 0.05 vs. baseline according to the T-Test for comparing the data of one group before and after treatment, § *p* ≤ 0.05 vs. point 1 according to the repeated-measures ANOVA with Dunnett post-hoc test.

To study their biological activity, the bradykinin-potentiating peptides Y-BPP and R-BPP were prepared by peptide synthesis and purified by reverse-phase chromatography. The potentiating effect was investigated in vivo in SD rats; arterial blood pressure was registered. Anesthetized rats received peptides through venous catheter, and the arterial catheter was used to record blood pressure (BP). Injection of bradykinin at a dose of 7 μg/kg resulted in the BP drop by 27–33% (Figure 8). Administration of either Y-BPP or R-BPP 10 min later produced a further small decrease (by about 10%) in BP and strongly enhanced the effect of bradykinin injected 5 min after BPP (Figure 8). The potentiating effect was dose-dependent (Figure 9) and was observed for about half an hour at the dose of 300 µg/kg and 1 mg/kg for Y-BPP (Figure 9A,B) and at the dose of 1 mg/kg for R-BPP (Figure 9C,D). No potentiating effect was detected one hour after BPP administration (Figure 9).

## 4. Discussion

Azemiopinae is regarded as a sister group of the Crotalinae [5,6]. Therefore, it is quite reasonable that the *A. feae* venom composition bears a resemblance to those of Crotalinae snakes. Thus, the main constituents of Feae’s viper venom are SVSPs (44.8%) and PLA2s (25.8%), representing about ¾ of venom total protein, although the content of SVMPs is fairly small as compared to other venoms of Crotalinae snakes [4]. The most remarkable difference between the Feae’s viper venom and those of other Crotalids is the presence of the peptide neurotoxin azemiopsin and 3FTxs in *A. feae* venom. Azemiopsin is homologous to waglerin from the Asian pit-viper *Tropidolaemus wagleri* venom but in contrast to waglerin has no disulfide bridge [15,32]. The content of azemiopsin in the venom is lower (3%) than that of waglerin (15% [33] or 38.2% [34]). Nevertheless, some neurological symptoms were observed in the victims of Feae’s viper bite [35], which might be caused by azemiopsin. Moreover, recently it was shown that *A. feae* venom interacted with fragments representing modified orthosteric mimotopes of binding region of nicotinic acetylcholine receptors from a diversity of potential preys [36]. This may also indicate the neurotoxicity of the *A. feae* venom. Indeed, inhibition of the muscle nAChRs was detected for azemiopsin [15], which later in preclinical studies was shown to be an efficient myorelaxant [26].

3FTxs are the main components of venoms from Elapidae snakes. They manifest a wide array of activities ranging from highly specific interactions with some receptors and ion channels to unspecific damage of cell membranes. 3FTxs were classified into several groups depending on their amino acid sequences and biological activities [37]. The most abundant groups are neurotoxins and cytotoxins (cardiotoxins). Cytotoxin-like components were reported in *Daboia russelii russelii* venom [38]. Transcripts of 3FTxs were identified in transcriptomes of several vipers, including *A. feae* [14]. However, no 3FTx neurotoxins were found in viper venoms so far. In the Feae’s viper venom, we have identified several 3FTxs (Figure 7); all these toxins contain the fifth disulfide bridge in the loop I and thus belong to so-called basal or non-conventional toxins [29,30]. Toxins of this group, similarly to α-neurotoxins, interact with some types of nicotinic acetylcholine receptors [39,40] and bind to muscarinic acetylcholine receptors as well [41,42]. It should be mentioned that the sequences of 3FTxs determined in this work are different from 3FTx-Aze-1 [14] described earlier. The differences between the published sequence 3FTx-Aze-1 and our sequence 3FTx-Aze-2 are not very big, including only four substitutions at positions 42, 63, 68 and 69, and may be explained by individual venom variations (Figure 5), while the other toxin amino acid identified in this work differs more strongly from that of 3FTx-Aze-1. The sequences of *A. feae* 3FTxs are distinguished by the number of amino acid residues between invariant cysteine residues. In addition, 3FTx-Aze-4 has an extended C-terminal tail, which makes it similar to long-type α-neurotoxins from elapid venoms. Based on the results obtained, it can be concluded that the *A. feae* 3FTxs are quite remarkable. The amino acid sequences of some *A. feae* 3FTxs have similarities with sequences of toxins from Colubridae. Interestingly, heterogeneity in sequences is observed within one species, and some of the sequences are closer to those from Viperidae and some from Colubridae. The first step to investigate the biological activity of *A. feae* three-finger toxins is our recent work, where the gene encoding the sequence of 3FTx-Aze-2 (Figure 5) was synthesized, expressed in *Escherichia. coli*, and the biological activity studies showed that the heterologously expressed toxin inhibited nicotinic acetylcholine receptors of both muscle and neuronal types [31].

It should be noted that the important components of Crotalidae venoms are bradykinin-potentiating peptides (BPP). BPPs are produced in venom glands by processing long precursor proteins including as a rule several sequences of BPP and natriuretic peptide. During mass-spectrometric analysis of *A. feae* venom, we have found two peptides, Y-BPP and R-BPP, with amino acid sequences homologous to BPPs (Figure 7). The synthetic BPPs showed bradykinin-potentiating effects (Figure 8 and Figure 9). Analysis of transcriptomic data revealed a nucleotide sequence encoding a polypeptide containing several azemiopsin sequences as well as sequences of both Y-BPP and R-BPP (Figure 5). We have not obtained precursors with sequence length similar to the ones determined before [13]; however, the shorter fragments fit well to the long precursor. It should be mentioned that the sequences of one BPP are different; the lysine residue in the long precursor is replaced by arginine in the sequence of R-BPP (Figure 6). The tripeptide fragment QKW is also found in the sequences of precursors. The tripeptide ZKW characteristic to Viperidae venoms is an endogenous inhibitor of metalloproteinases [43,44]. Usually, several QKW repeats have been found in the CNP precursors; however, only one is found in sequence from *A. feae*.

In addition, if we fully cover the repertoire of toxins from what we found only in the form of transcripts, it turns out that *A. feae* retained a wider range of toxins from the common ancestor of Viperidae. Apparently, the common ancestor had a much wider arsenal of toxins. It is logical to assume that retaining a wider range of toxins is due to the absence of strict selection in the habitat of *A. feae*; in other Viperidae, the selection was apparently due to specialization in prey and a change in living conditions.

For the Viperidae, an origin in Asia was presumed, and for the Crotalinae (and Azemiops), the origin was firmly placed in Asia [5,6]. It can be assumed that *A. feae* is a relic (“ancient old-timer”) whose habitat was stable enough to leave the entire toxin repertoire or at least part of it almost unchanged. Various assumptions can be made about the presence of transcripts, but the lack of protein would indicate that this is just a blank transcription. Again, the lack of selection minimizes their divergence. The found unique peptides may be just a new type of adaptation and a unique feature of *A. feae* or further development of toxins inherited from an ancient ancestor.

For biological activity studies, Y-BPP and R-BPP were prepared by solid phase synthesis. Studies on SD rats revealed that both peptides dose-dependently potentiated the activity of bradykinin. At 1 mg/kg, the peptides increased the bradykinin effects by 30% (Figure 9B,D). This value is similar to the ones observed for some BPPs from *Bothrops* venoms [45]. While Y-BPP has some structural similarity to BPPs earlier described, for example to BPPB from *Agkistrodon blomhofii* (Figure 7), the sequence of R-BPP is unique. Thus, we have found a BPP with a new structural motif.

## 5. Conclusions

We have described the comprehensive venom-gland transcriptomic and quantitative proteomic venom characterization of *A. feae* viper. The proteomic analysis revealed the presence of 120 unique proteins, serine proteinases and phospholipases A_2_ being the most abundant. In total, toxins representing 14 families were identified, among which bradykinin-potentiating peptides with unique amino acid sequence features were found and shown to possess biological activity in vivo. The proteomic analysis also revealed three-finger toxins which belong to the group of non-conventional toxins possessing neurotoxic activity. This is the first indication of the presence of three-finger neurotoxins in viper venom. The transcriptomic analysis of the venom gland by Illumina next-generation sequencing revealed 206 putative venom transcripts. Together, the study unveiled the venom proteome and venom gland transciptome of *A. feae,* which in general resemble those of snakes from the Viperidae family. However, new toxins not found earlier in viper venom and including three-finger toxins and unusual bradykinin-potentiating peptides were discovered.

## Supplementary Materials

The following are available online at https://www.mdpi.com/2227-9059/8/8/249/s1, Figure S1: Gene ontology by categories of biological process, cellular component and molecular function, Figure S2: Functional categories of the contigs in KOG database. (A), RNA processing and modification; (B), chromatin structure and dynamics; (C), energy production and conversion; (D), cell cycle control, cell division, chromosome partitioning; (E), amino acid transport and metabolism; (F), nucleotide transport and metabolism; (G), carbohydrate transport and metabolism; (H), coenzyme transport and metabolism; (I), lipid transport and metabolism; (J), translation, ribosomal structure and biogenesis; (K), transcription; (L), replication, recombination and repair; (M), cell wall/membrane/envelope biogenesis; (N), cell motility; (O), posttranslational modification, protein turnover, chaperones; (P), inorganic ion transport and metabolism; (Q), secondary metabolites biosynthesis, transport and catabolism; (R), general function prediction only; (T), signal transduction mechanisms; (U), intracellular trafficking, secretion and vesicular transport; (V), defense mechanisms; (W), extracellular structures; (Y), nuclear structure; (Z), cytoskeleton; (S), function unknown, Figure S3: The distribution of the top 10 BLAST hits. Snake species are underlined, Figure S4: (A) Multiple sequence alignment of serine proteases (SVSPs) from the venom gland transcriptome of *A. feae* in comparison with the described serine proteases from *A. feae*. Alignment generated by MUSCLE (Here and in all following captions, MUSCLE is MUltiple Sequence Comparison by Log-Expectation) with percentage identity coloring scheme. (B) Multiple sequence alignment overview window, for serine proteases (SVSPs) from the venom gland transcriptome of *A. feae* in comparison with the described serine proteases from *A. feae*. Alignment generated by MUSCLE (Here and in all following captions, MUSCLE is MUltiple Sequence Comparison by Log-Expectation) with ClustalX Colour Scheme, Figure S5: (A) Multiple sequence alignment of phospholipase A2 (PLA2) transcripts from the venom gland transcriptome of *A. feae* in comparison to PLA2 sequences of representative venomous snakes. Amino acids that determine the type of phospholipase (D49, N49 or K49) are marked with a black frame and a red font. Alignment generated by MUSCLE with ClustalX Colour Scheme. (B) Neighbor-joining (NJ) gene tree, based on BLOSUM62 matrix alignment of the proteins in the PLA2 cluster. Branches corresponding to acidic phospholipases are marked in yellow; branches corresponding to basic phospholipases are marked in blue. New discovered isoforms are marked in red. PLA2 previously discovered by other researchers are marked in grey boxes. Human PLA2 was used as outgroup, Figure S6: Multiple sequence alignment of L-amino-acid oxidase (LAAO) transcripts from *A. feae* with two Viperidae homologs. Alignment generated by MUSCLE with ClustalX Colour Scheme, Figure S7: (A) Multiple sequence alignment of zinc metalloprotease (SVMP) transcripts from *A. feae* in comparison to zinc metalloprotease sequences of representative venomous snakes. Alignment generated by MUSCLE with percentage identity coloring scheme. (B) Multiple sequence alignment overview window for zinc metalloprotease (SVMP) transcripts from *A. feae* in comparison to zinc metalloprotease sequences of representative venomous snakes. The top of the alignment shows the domain organization of the reference metalloproteinases. Alignment generated by MUSCLE with ClustalX Colour Scheme, Figure S8: Multiple sequence alignment of snake venom Vascular Endothelial Growth Factors (svVEGF) in comparison to svVEGF sequences of representative venomous snakes. Alignment is separated by bold lines into blocks according to types of svVEGF. Alignment generated by MUSCLE with ClustalX Colour Scheme, Figure S9: Multiple sequence alignment of phospholipase B (PLB) transcripts from the venom gland transcriptome of *A. feae* in comparison to PLB sequences of representative venomous snakes. Alignment generated by MUSCLE with percentage identity coloring scheme, Figure S10: Multiple sequence alignment of venom phosphodiesterase 1 (vPDE) transcripts from the venom gland transcriptome of *A. feae* in comparison to vPDE sequences of representative venomous snakes. Alignment generated by MUSCLE with ClustalX Colour Scheme, Figure S11: Multiple sequence alignment of hyaluronidases (HYA) with *Protobothrops mucrosquamatus* homolog. Alignment generated by MUSCLE with ClustalX Colour Scheme, Figure S12: Multiple sequence alignment of nerve growth factor (svNGF) partial transcripts from *A. feae* in comparison to svNGF sequences of representative venomous snakes. Alignment generated by MUSCLE with ClustalX Colour Scheme, Figure S13: Multiple sequence alignment of cystatins (CST) from the venom gland transcriptome of *A. feae* in comparison to cystatins sequences of representative venomous snakes. Alignment is separated by bold lines into blocks according to types of cystatins. Alignment generated by MUSCLE with ClustalX Colour Scheme, Figure S14: Multiple sequence alignment of kazal (KAZ) domains with comparison to kazal-like sequences of representative venomous snakes. Alignment generated by MUSCLE with ClustalX Colour Scheme, Figure S15: Multiple sequence alignment of acetylcholinesterase (AChE) partial transcripts from the venom gland transcriptome of *A. feae* with two *Vipera anatolica cholinesterases*. Alignment generated by MUSCLE with percentage identity coloring scheme, Figure S16: Multiple sequence alignment of calcium-binding protein (Reticulocalbin) transcripts in comparison to sequences of representative venomous snakes. Alignment generated by MUSCLE with ClustalX Colour Scheme, Figure S17: Multiple sequence alignment of translationally controlled tumor protein (TCTP/HRF) transcripts from the venom gland transcriptome of *A. feae* in comparison with *Crotalus horridus* homolog. Alignment generated by MUSCLE with percentage identity coloring scheme, Figure S18: Multiple sequence alignment of lysosomal acid phosphatase (vAP) from the venom gland transcriptome of *A. feae* in comparison to vAP sequences of representative venomous snakes. Alignment generated by MUSCLE with percentage identity coloring scheme, Figure S19: Sequence alignment of insulin-like growth factor-binding protein (IGFBP) partial transcript from the venom gland transcriptome of *A. feae* in comparison with *Protobothrops mucrosquamatus* homolog. Alignment generated by MUSCLE with percentage identity coloring scheme, Figure S20: Multiple sequence alignment of venom prothrombin activator (vPA) partial transcripts from the venom gland transcriptome of *A. feae* in comparison with *Oxyuranus microlepidotus* homolog. Alignment generated by MUSCLE with percentage identity coloring scheme. Figure S21: Multiple sequence alignment of kunitz (KUN) domains with comparison to kunitz-like sequences of representative venomous snakes. Alignment generated by MUSCLE with percentage identity coloring scheme, Figure S22: Multiple sequence alignment of glutaminyl-peptide cyclotransferase (QC) partial transcripts domains with *Protobothrops mucrosquamatus* homolog. Alignment generated by MUSCLE with percentage identity coloring scheme, Figure S23: Multiple sequence alignment of dipeptidyl peptidase (vDP) transcripts in comparison with *Protobothrops mucrosquamatus* homologs. Alignment generated by MUSCLE with ClustalX Colour Scheme, Figure S24: Multiple sequence alignment of 5′-nucleotidase (NUC) from the venom gland transcriptome of *A. feae* in comparison to 5′-nucleotidase sequences of representative venomous snakes. Alignment generated by MUSCLE with ClustalX Colour Scheme, Figure S25: Multiple sequence alignment of C-type lectins with other snaclecs. Alignment generated by MUSCLE with percentage identity coloring scheme, Figure S26: Multiple sequence alignment of fibrinogen-like partial transcripts from the venom gland transcriptome of *A. feae* in comparison to fibrinogen-like sequences of representative venomous snakes. Alignment generated by MUSCLE with percentage identity coloring scheme, Figure S27: Multiple sequence alignment serine carboxypeptidase (vSC) partial transcripts with *Protobothrops mucrosquamatus* homolog. Alignment generated by MUSCLE with percentage identity coloring scheme, Figure S28: Multiple sequence alignment of waprin transcripts in comparison to waprin sequences of representative venomous snakes. Alignment generated by MUSCLE with ClustalX Colour Scheme, Figure S29: Sections of MS/MS spectra of azemiopsin and ist N-terminally extended analogs; shift of the b-ions (azemiopsin 416 = b3, DNW; Ser-azemiopsin 503 = b4, SDNW; Glu-Ser-Azemiopsin, 632 = b5, ESDNW), Figure S30: MS/MS spectrum of R-BPP and its interpretation; the insert shows the theoretical b- and y-ions for the sequence ZRPPGVYYPP, with Z for N-terminal pyroglutamic acid (theoretical mass 1156.579); strong internal ions are observed due to preferential fragmentation at Pro, Table S1: Qualitative proteomic data, Table S2: Library basic statistics, Table S3: Transcriptome assembly statistics (Trinity), Table S4: Putative venom transcripts, Table S5: Peptidome of *A. feae* venom.
