# Supplementary material for: Novel Bradykinin-Potentiating Peptides and Three-Finger Toxins from Viper Venom: Combined NGS Venom Gland Transcriptomics and Quantitative Venom Proteomics of the Azemiops feae Viper
Source: Biomedicines. 2020 Jul 28;8(8):249. doi: 10.3390/biomedicines8080249 (PMC7460416; doi:10.3390/biomedicines8080249)
Supplement: Supplementary file 1 [file biomedicines-08-00249-s001.zip › Supplementary materials/Table S3.docx]

**Table S3.** Transcriptome assembly statistics (Trinity)

| **Counts of transcripts TSA id (in progress)** | |
| --- | --- |
| Total trinity 'genes' | 18,296 |
| Total trinity transcripts | 20,085 |
| Percent GC | 43.08 |
| **Stats based on ALL transcript contigs** | |
| Contig N50 | 781 |
| Median contig length | 392 |
| Average contig | 599 |
| Total assembled bases | 12,022,068 |
| **Stats based on ONLY LONGEST ISOFORM per 'GENE'** | |
| Contig N50 | 687 |
| Median contig length | 373 |
| Average contig | 556 |
| Total assembled bases | 10,176,020 |
