# Supplementary material for: Novel Bradykinin-Potentiating Peptides and Three-Finger Toxins from Viper Venom: Combined NGS Venom Gland Transcriptomics and Quantitative Venom Proteomics of the Azemiops feae Viper
Source: Biomedicines. 2020 Jul 28;8(8):249. doi: 10.3390/biomedicines8080249 (PMC7460416; doi:10.3390/biomedicines8080249)
Supplement: Supplementary file 1 [file biomedicines-08-00249-s001.zip › Supplementary materials/Tables S2.docx]

**Table S2.** Library basic statistics.

| **species** | **sample type** | **nucleotides** | **Total reads** | **%GC** | **mean read length** | **BioProject ID** |
| --- | --- | --- | --- | --- | --- | --- |
| *Azemiops feae* | venom gland | 2,092,030,920 | 29,055,985 | 49,5 | 72 | PRJNA504599 |
