# Supplementary material for: Novel Bradykinin-Potentiating Peptides and Three-Finger Toxins from Viper Venom: Combined NGS Venom Gland Transcriptomics and Quantitative Venom Proteomics of the Azemiops feae Viper
Source: Biomedicines. 2020 Jul 28;8(8):249. doi: 10.3390/biomedicines8080249 (PMC7460416; doi:10.3390/biomedicines8080249)
Supplement: Supplementary file 1 [file biomedicines-08-00249-s001.zip › Supplementary materials/Figures S1-S30-V2.pdf]

Figures

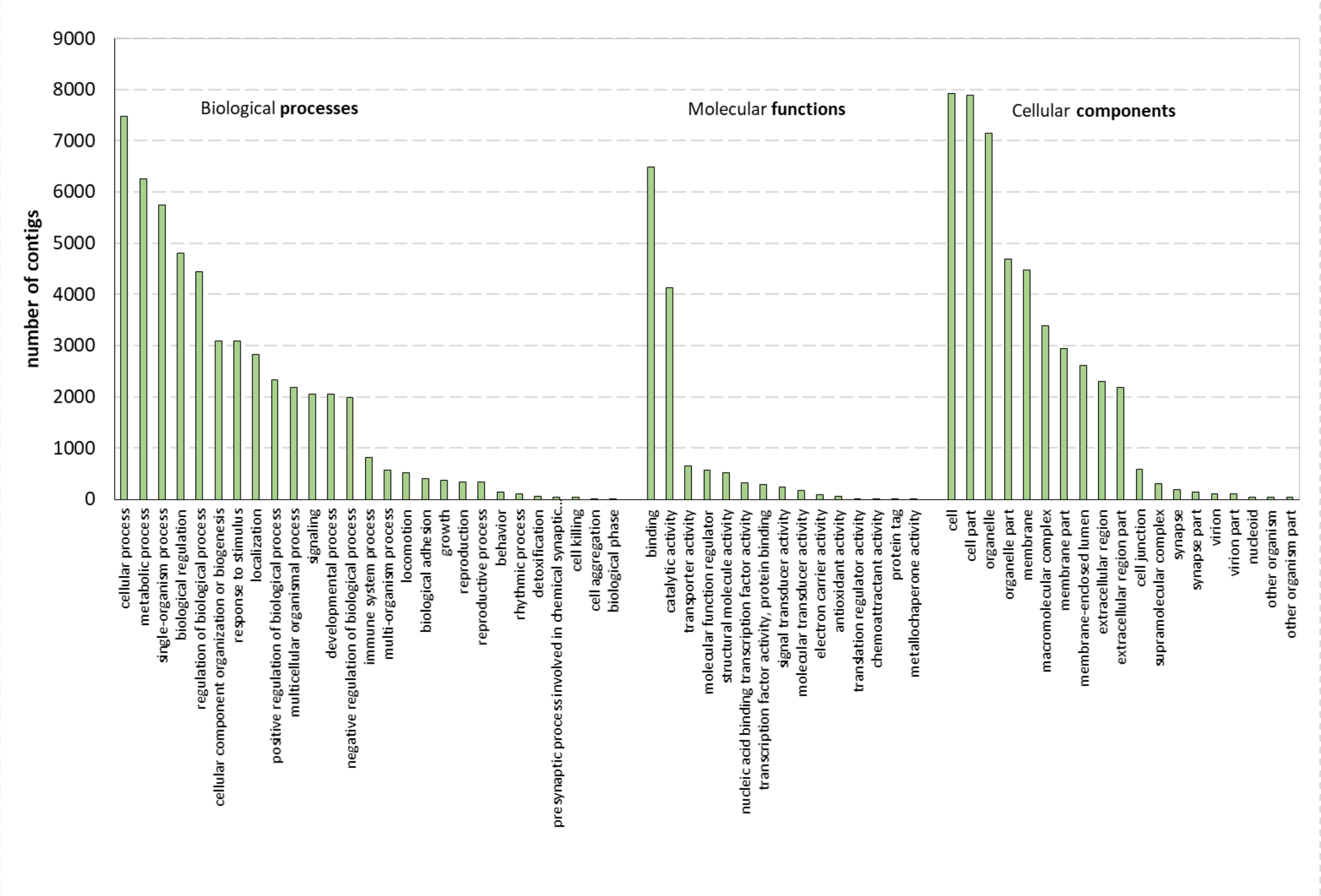

Figure S1. Gene ontology by categories of biological process, cellular component and molecular function.

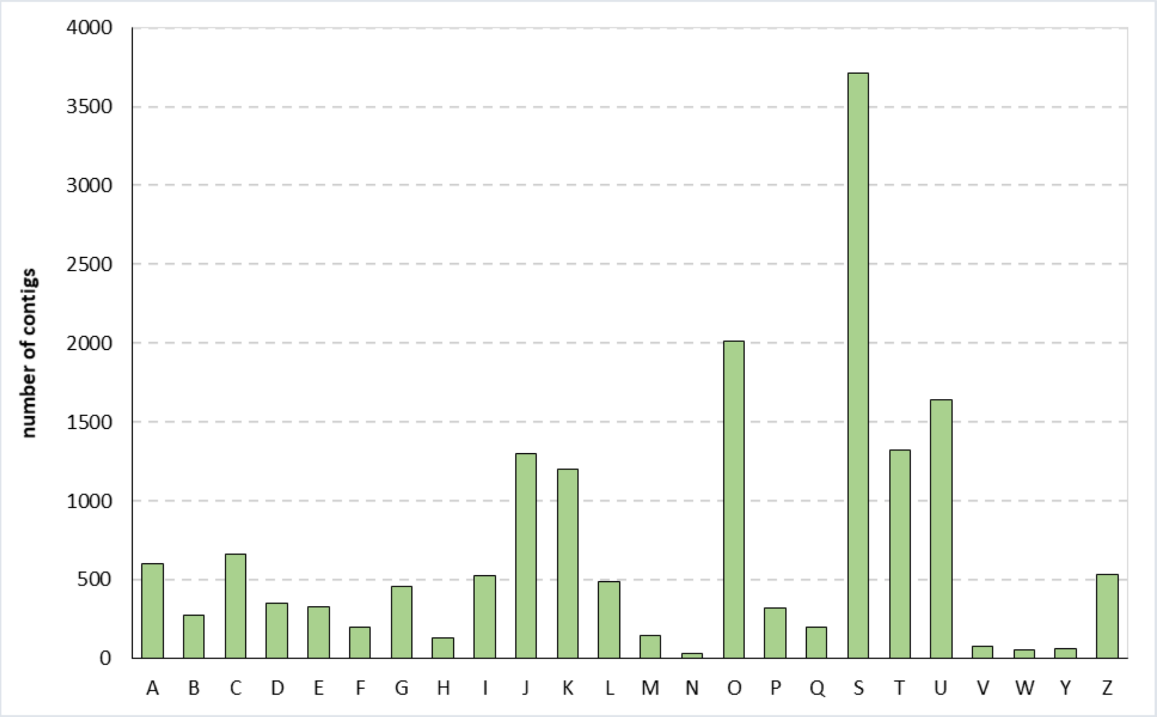

Figure S2. Functional categories of the contigs in KOG database. A, RNA processing and modification; B, chromatin structure and dynamics; C, energy production and conversion D, cell cycle control, cell division, chromosome partitioning; E, amino acid transport and metabolism; F, nucleotide transport and metabolism; G, carbohydrate transport and metabolism; H, coenzyme transport and metabolism; I, lipid transport and metabolism; J, translation, ribosomal structure and biogenesis; K, transcription; L, replication, recombination and repair; M, cell wall/membrane/envelope biogenesis; N, cell motility; O, posttranslational modification, protein turnover, chaperones; P, inorganic ion transport and metabolism; Q, secondary metabolites biosynthesis, transport and catabolism; R, general function prediction only; T, signal transduction mechanisms; U, intracellular trafficking, secretion, and vesicular transport; V, defense mechanisms; W, extracellular structures; Y, nuclear structure; Z, cytoskeleton; S, function unknown.

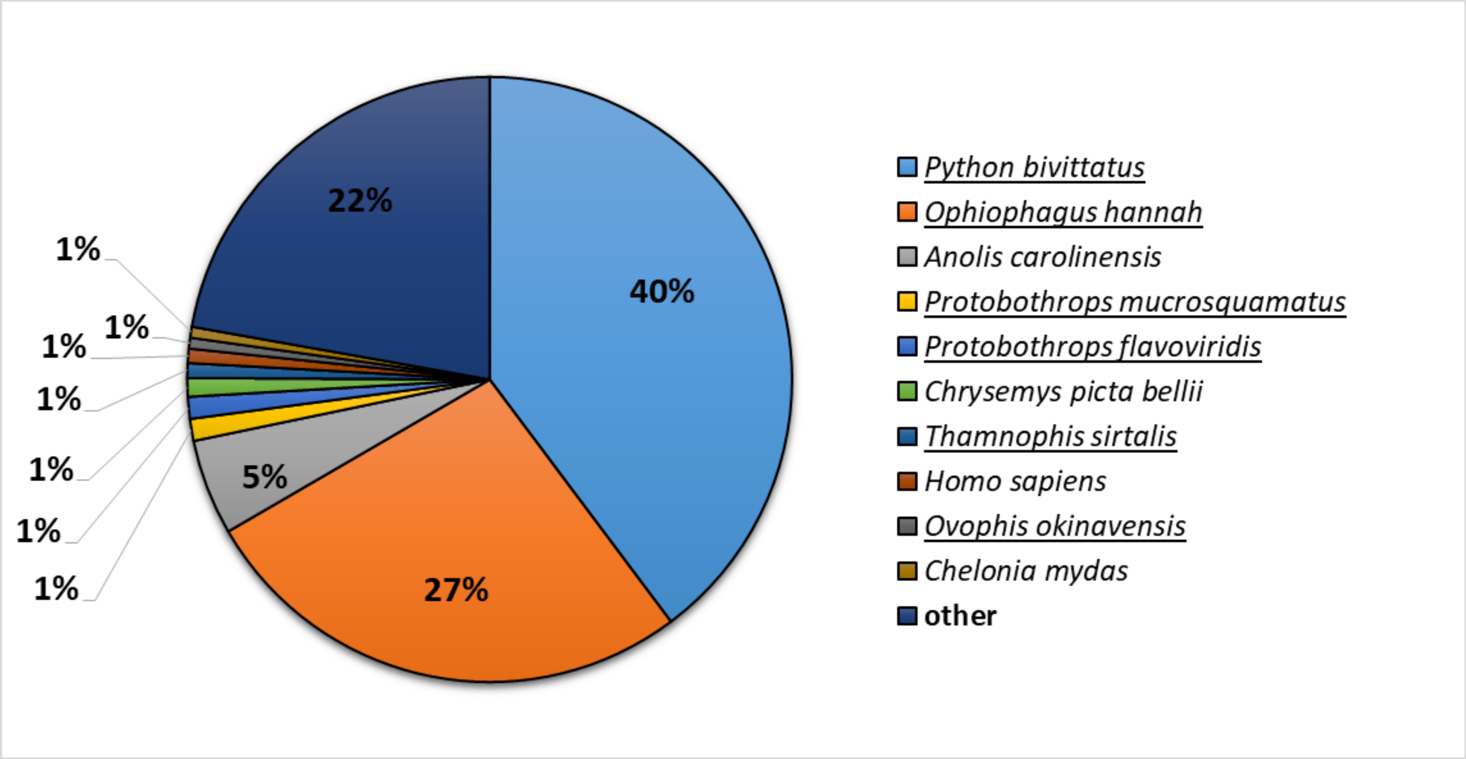

Figure S3. The distribution of the top 10 BLAST hits. Snake species are underlined.

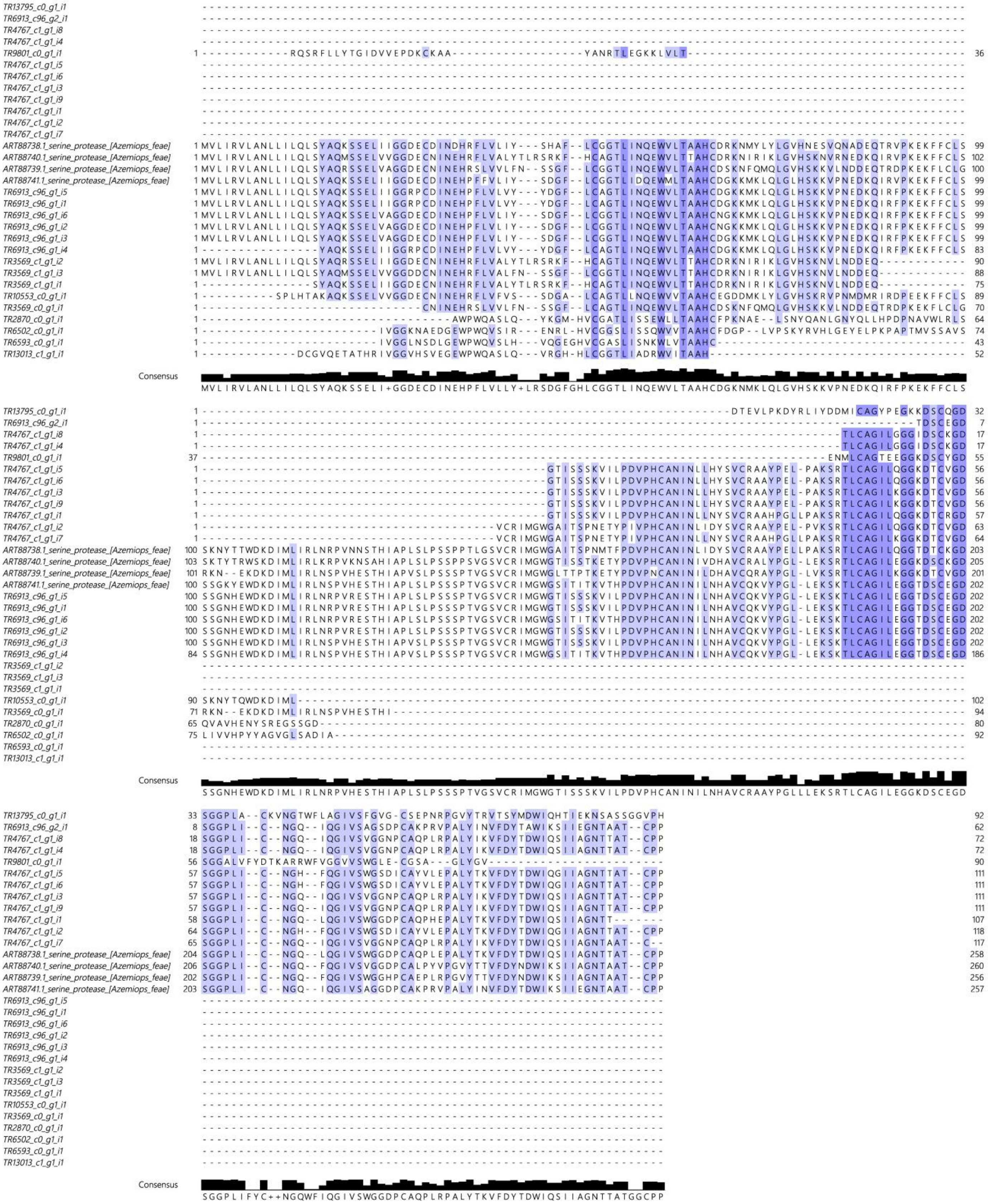

Figure S4. A) Multiple sequence alignment of **serine proteases** (SVSPs) from the venom gland transcriptome of *A. feae* in comparison with the described serine proteases from *A. feae*. Alignment generated by MUSCLE (Here and in all following captions MUSCLE is MUltiple Sequence Comparison by Log-Expectation) with percentage Identity coloring scheme.

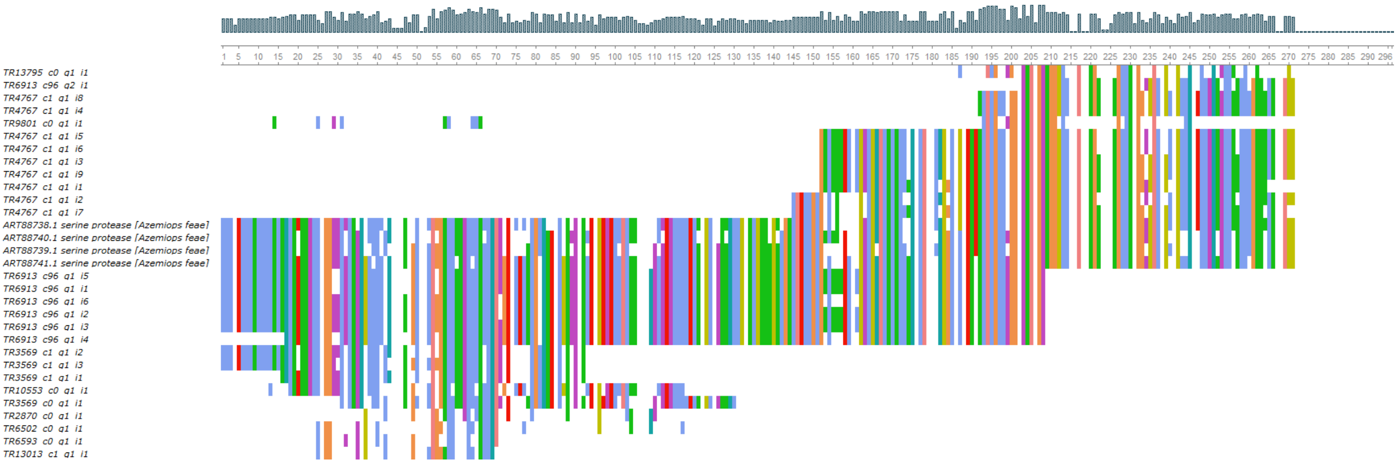

Figure S4. B) Multiple sequence alignment overview window, for serine proteases (SVSPs) from the venom gland transcriptome of A. feae in comparison with the described serine proteases from A. feae. Alignment generated by MUSCLE (Here and in all following captions MUSCLE is MUltiple Sequence Comparison by Log-Expectation) with ClustalX Colour Scheme.

A)

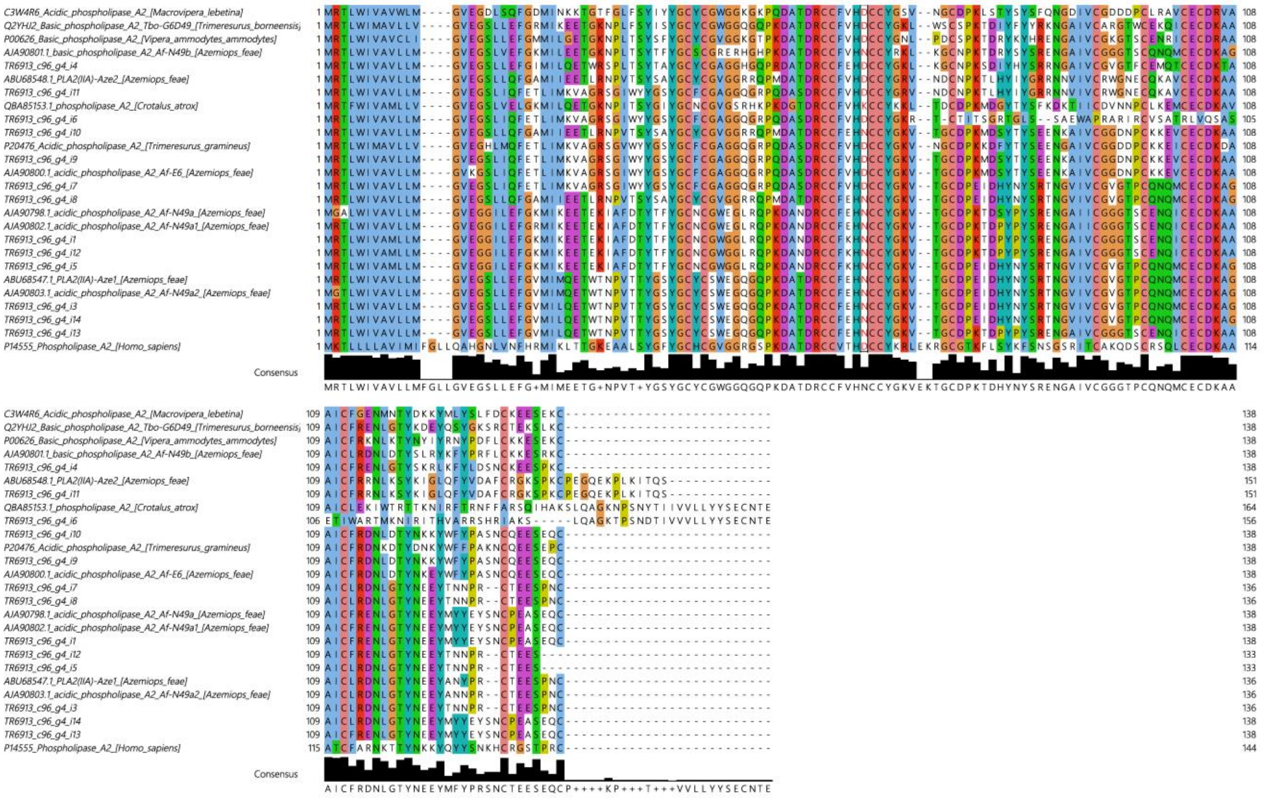

B)

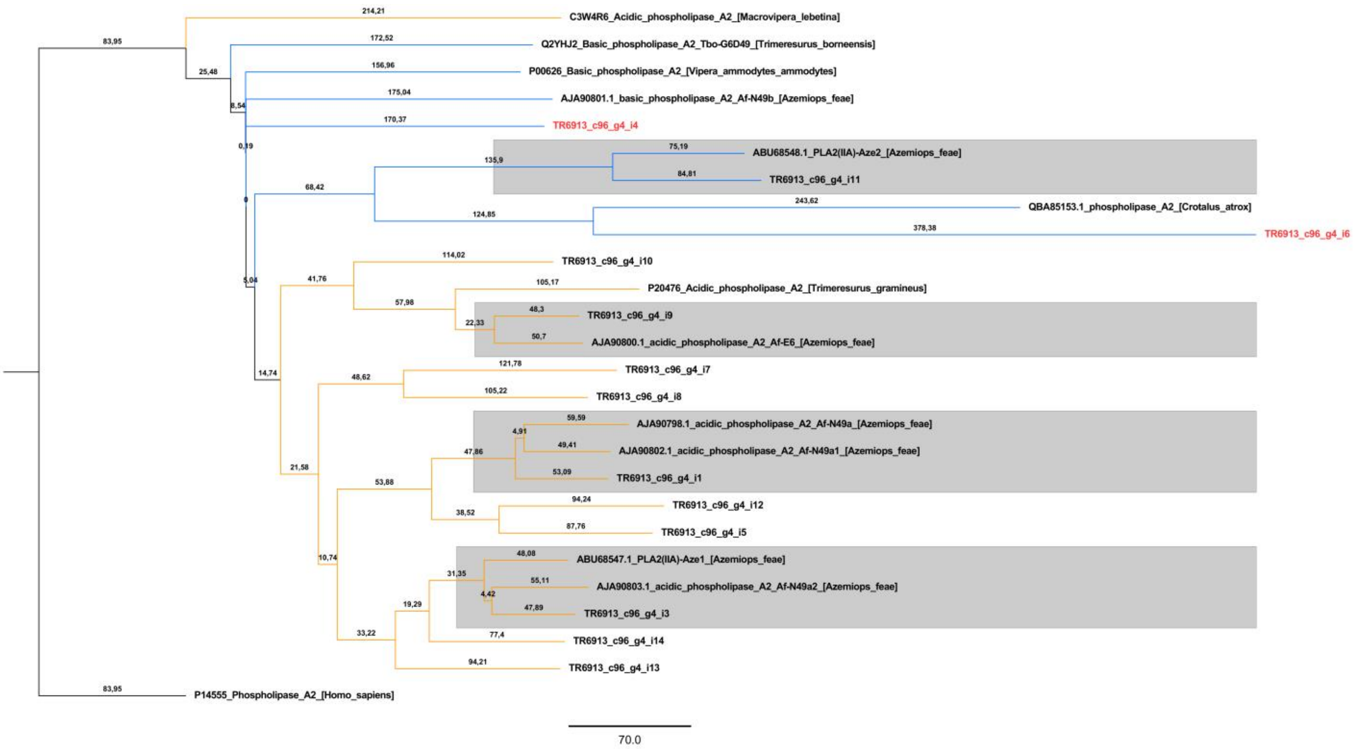

Figure S5. A) Multiple sequence alignment of **phospholipase A2** (PLA2) transcripts from the venom gland transcriptome of *A. feae* in comparison to PLA2 sequences of representative venomous snakes. Amino acids that determine the type of phospholipase (D49, N49 or K49) are marked with a black frame and a red font. Alignment generated by MUSCLE with ClustalX Colour Scheme. **B)** Neighbor joining (NJ) gene tree, based on BLOSUM62 matrix alignment of the proteins in the PLA2 cluster. Branches corresponding to acidic phospholipases are marked in yellow, branches corresponding to basic phospholipases are marked in blue. New discovered isoforms are marked in red. PLA2 previously discovered by other researchers are marked in grey boxes. Human PLA2 was used as outgroup.

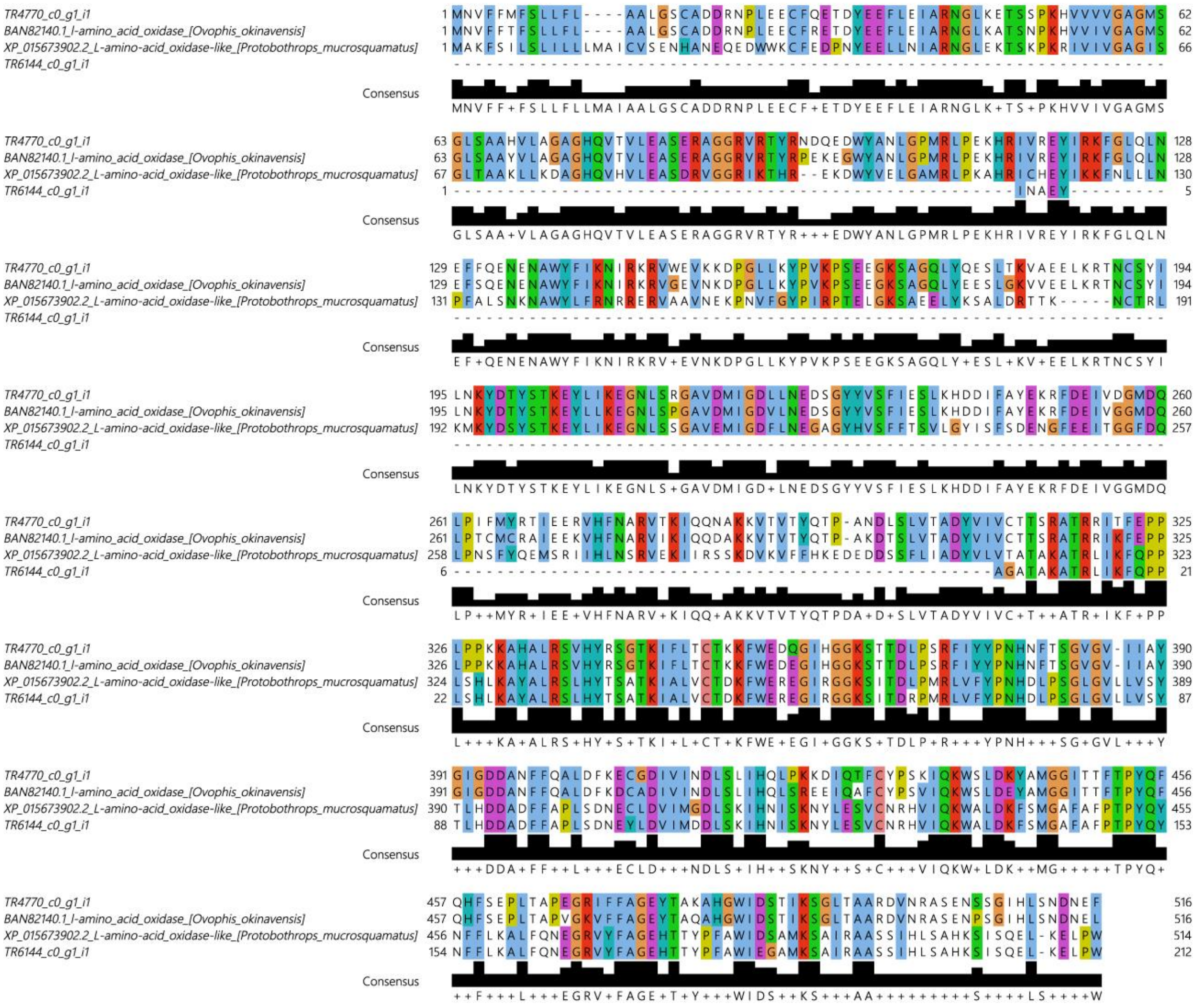

Figure S6. Multiple sequence alignment of **L-amino-acid oxidase** (LAAO) transcripts from *A. feae* with two *Viperidae* homologs. Alignment generated by MUSCLE with ClustalX Colour Scheme.

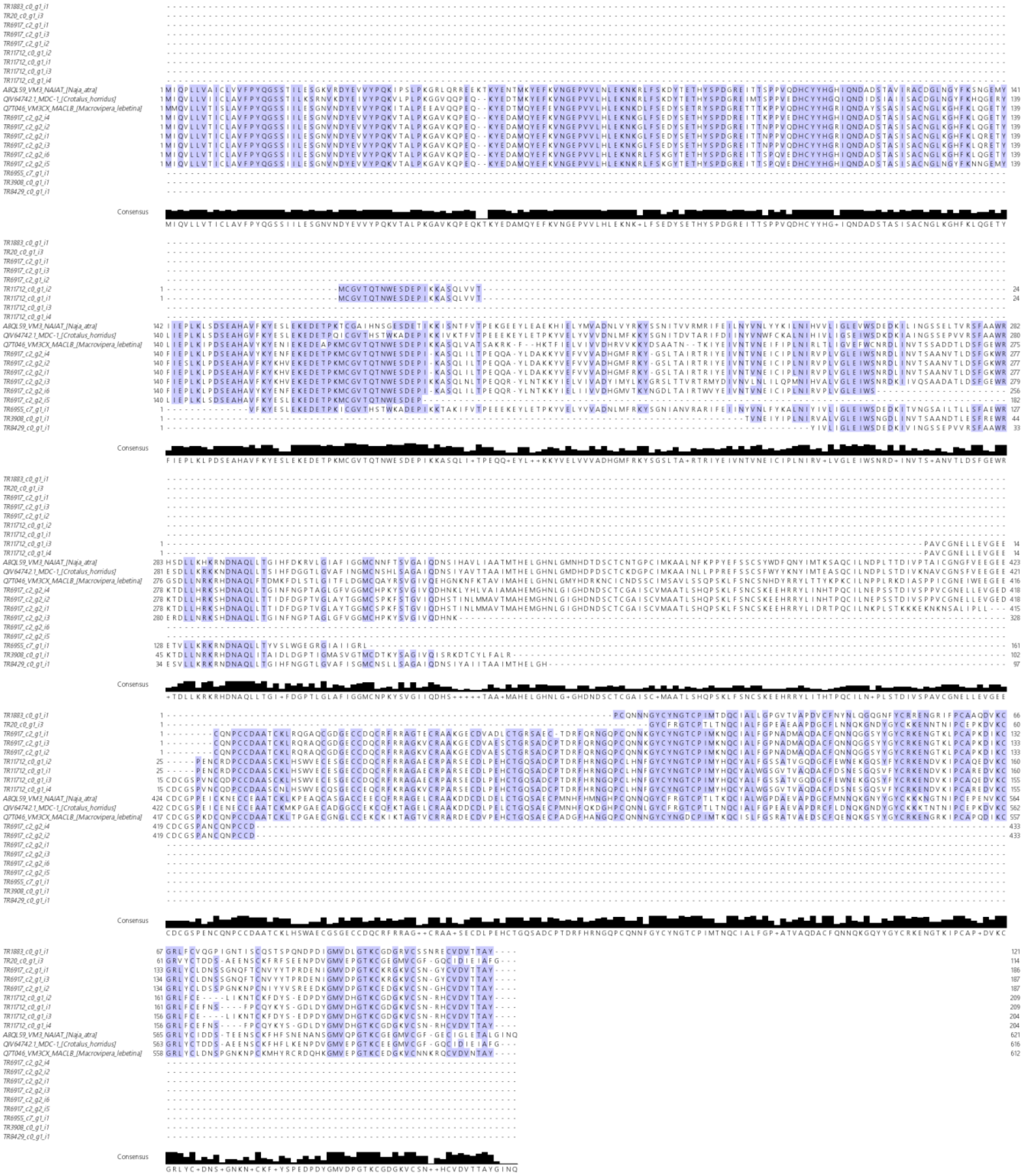

Figure S7. A) Multiple sequence alignment of **zinc metalloprotease** (SVMP) transcripts from *A. feae* in comparison to zinc metalloprotease sequences of representative venomous snakes. Alignment generated by MUSCLE with percentage Identity coloring scheme.

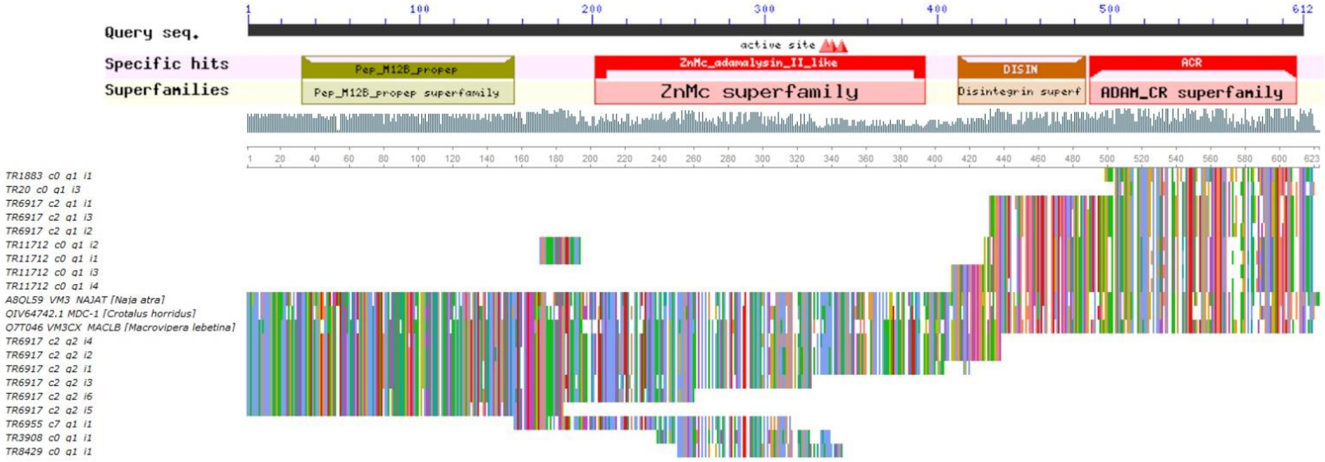

Figure S7. B) Multiple sequence alignment overview window, for zinc metalloprotease (SVMP) transcripts from A. feae in comparison to zinc metalloprotease sequences of representative venomous snakes. In the top of the alignment shows the domain organization of the reference metalloproteinases. Alignment generated by MUSCLE with ClustalX Colour Scheme.

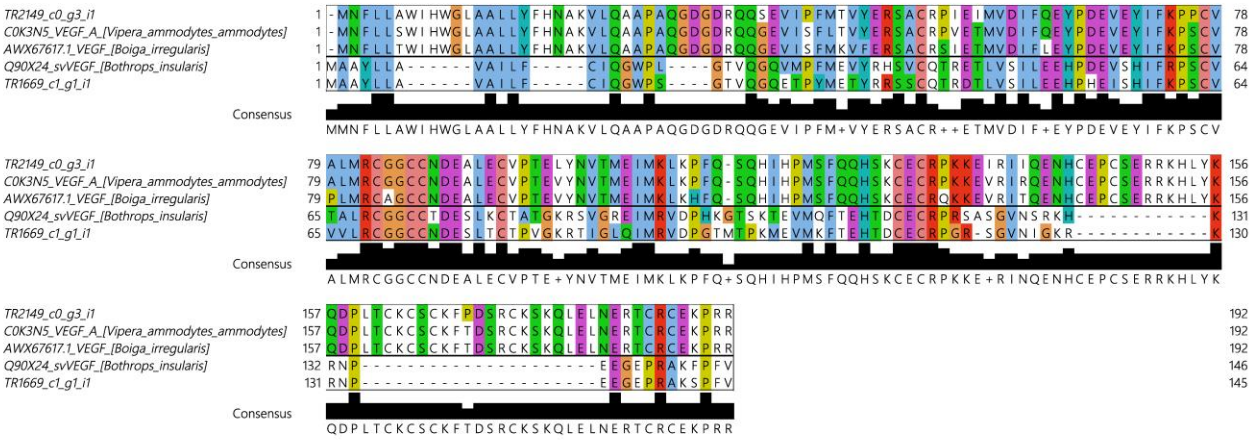

Figure S8. Multiple sequence alignment of **snake venom Vascular Endothelial Growth Factors** (svVEGF) in comparison to svVEGF sequences of representative venomous snakes. Alignment is separated by bold lines into blocks according to types of svVEGF. Alignment generated by MUSCLE with ClustalX Colour Scheme.

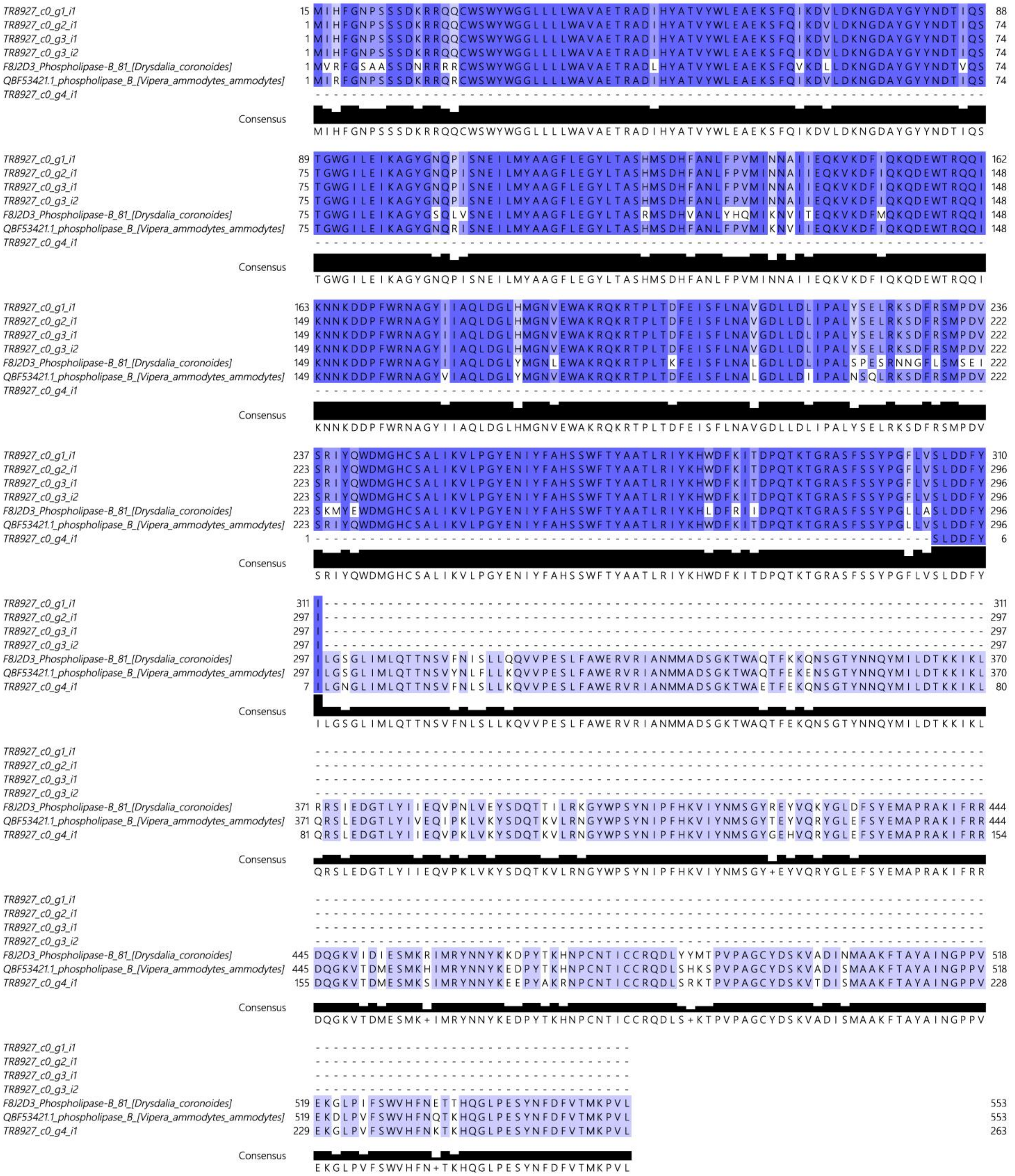

Figure S9. Multiple sequence alignment of **phospholipase B** (PLB) transcripts from the venom gland transcriptome of A. feae in comparison to PLB sequences of representative venomous snakes. Alignment generated by MUSCLE with percentage Identity coloring scheme.

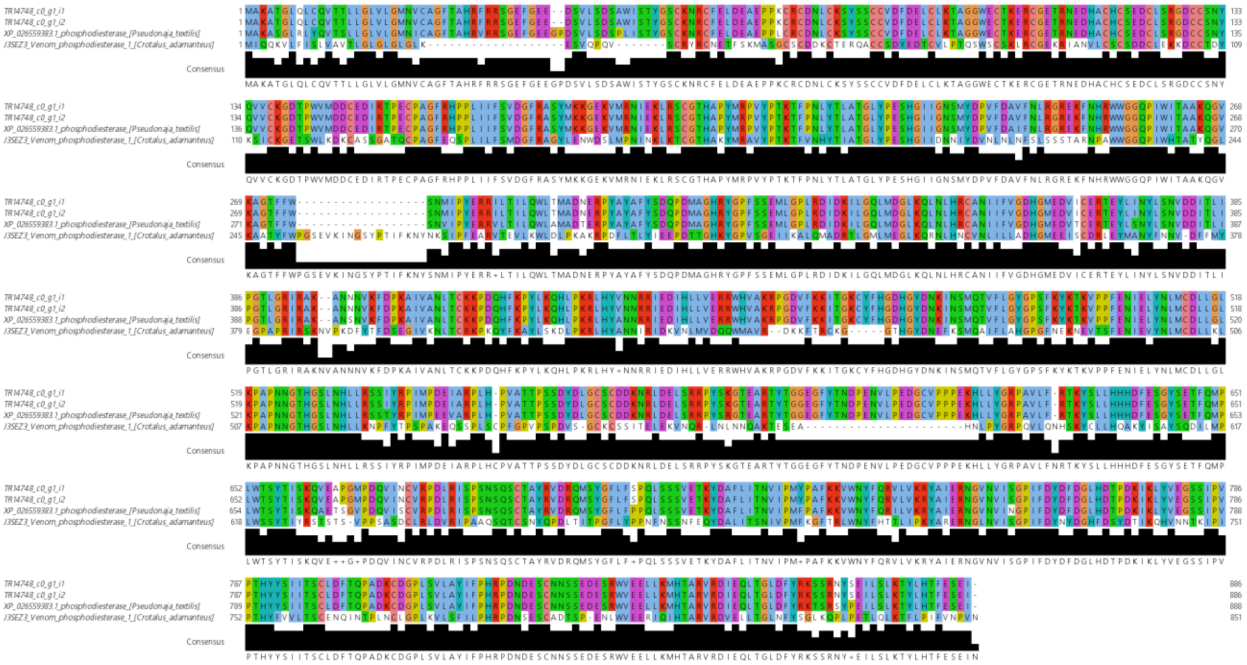

Figure S10. Multiple sequence alignment of **venom phosphodiesterase 1** (vPDE) transcripts from the venom gland transcriptome of A. feae in comparison to vPDE sequences of representative venomous snakes. Alignment generated by MUSCLE with ClustalX Colour Scheme.

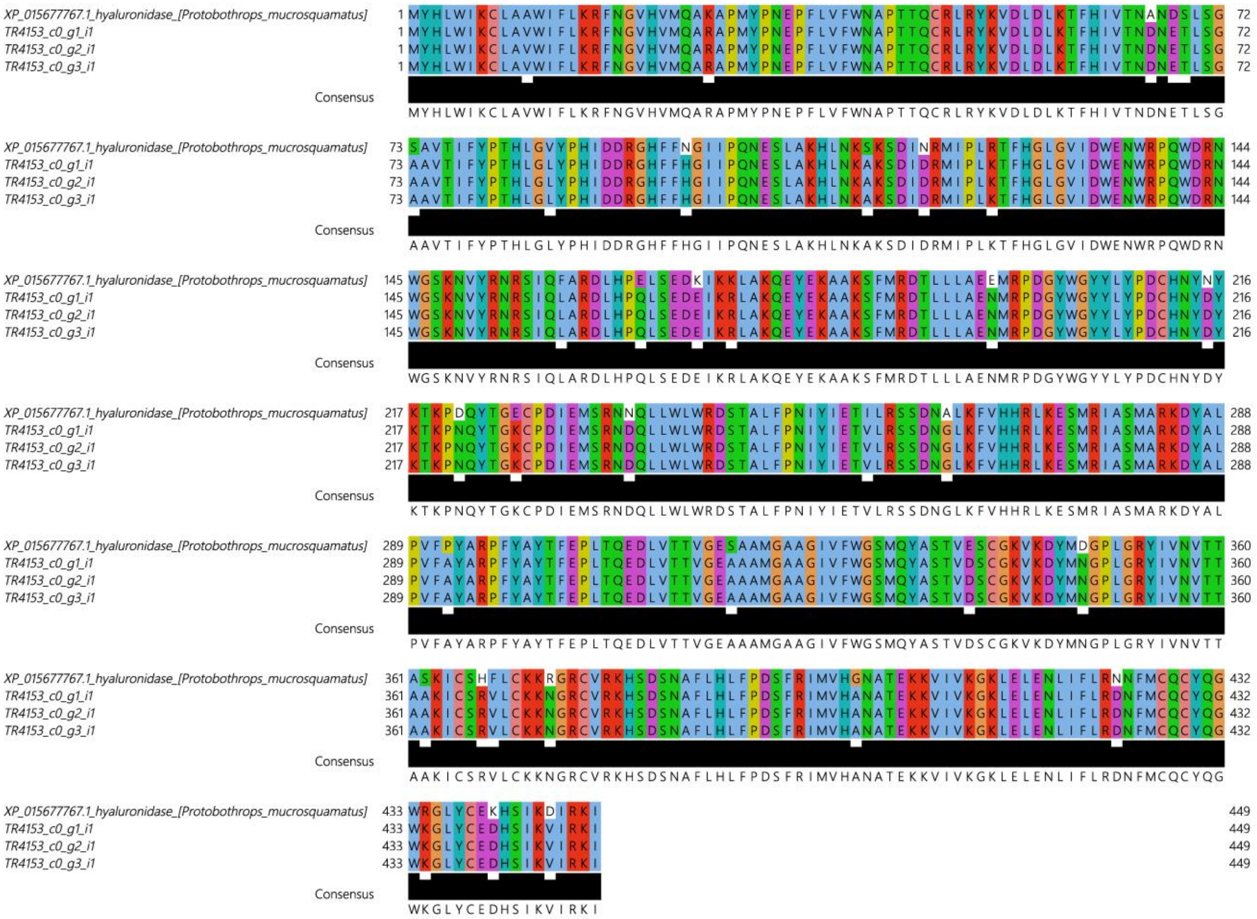

Figure S11. Multiple sequence alignment of **hyaluronidases** (HYA) with *Protobothrops mucrosquamatus* homolog. Alignment generated by MUSCLE with ClustalX Colour Scheme.

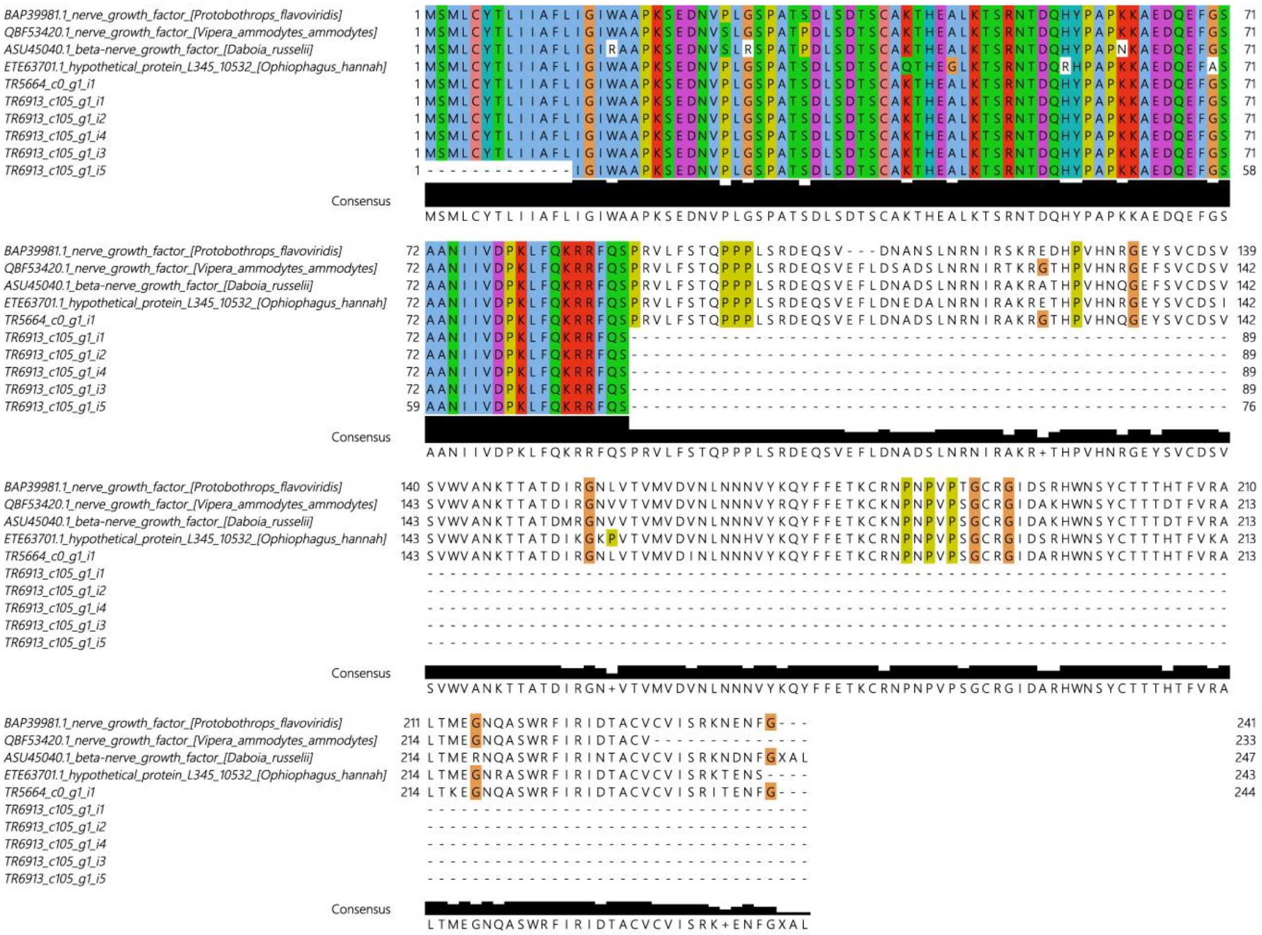

Figure S12. Multiple sequence alignment of **nerve growth factor** (svNGF) partial transcripts from *A. feae* in comparison to svNGF sequences of representative venomous snakes. Alignment generated by MUSCLE with ClustalX Colour Scheme.

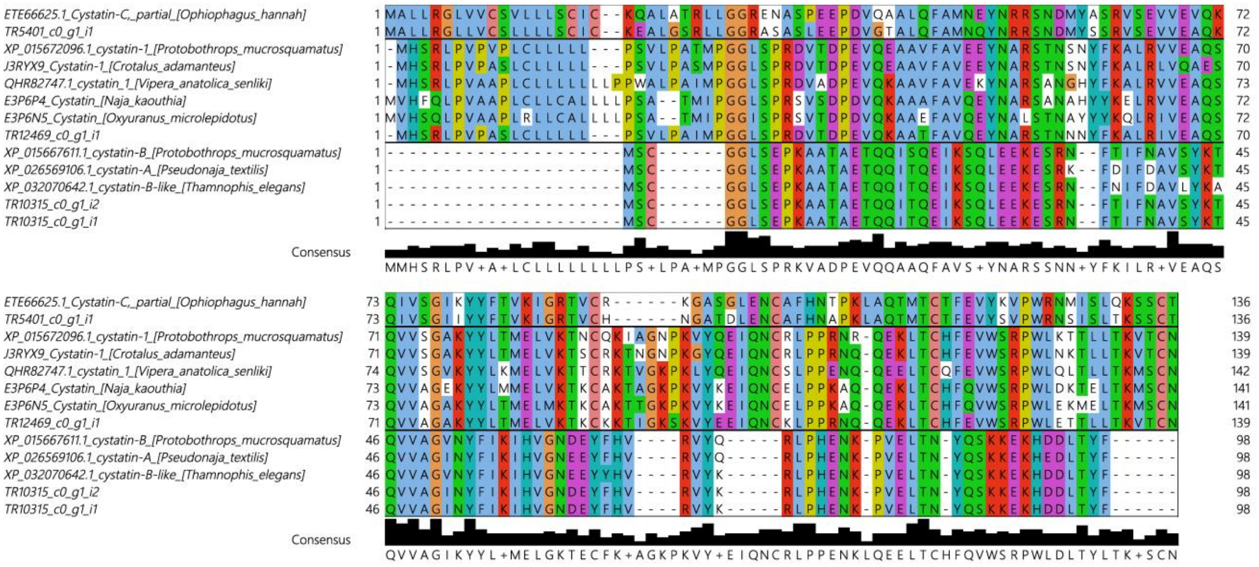

Figure S13. Multiple sequence alignment of **cystatins** (CST) from the venom gland transcriptome of *A. feae* in comparison to cystatins sequences of representative venomous snakes. Alignment is separated by bold lines into blocks according to types of cystatins. Alignment generated by MUSCLE with ClustalX Colour Scheme.

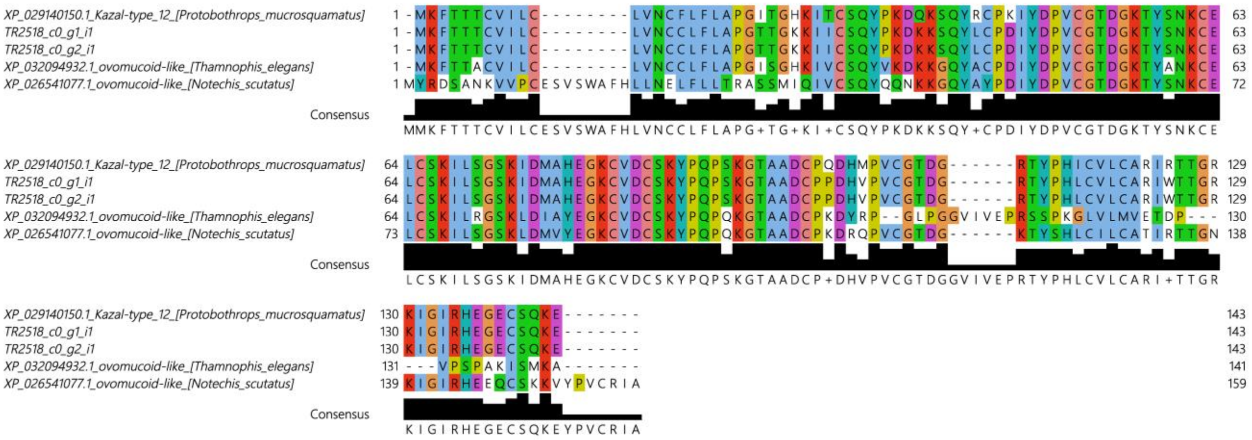

Figure S14. Multiple sequence alignment of **kazal** (KAZ) domains with comparison to kazal-like sequences of representative venomous snakes. Alignment generated by MUSCLE with ClustalX Colour Scheme.

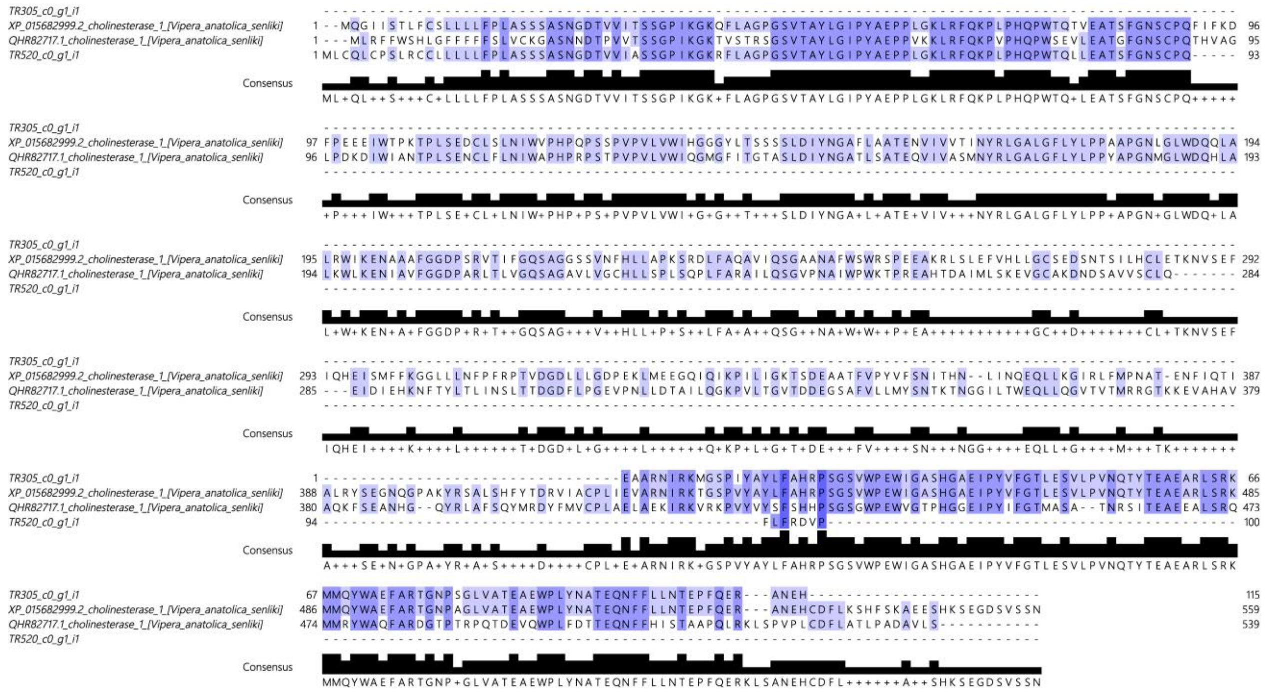

Figure S15. Multiple sequence alignment of **acetylcholinesterase** (AChE) partial transcripts from the venom gland transcriptome of *A. feae* with two *Vipera anatolica* cholinesterases. Alignment generated by MUSCLE with percentage Identity coloring scheme.

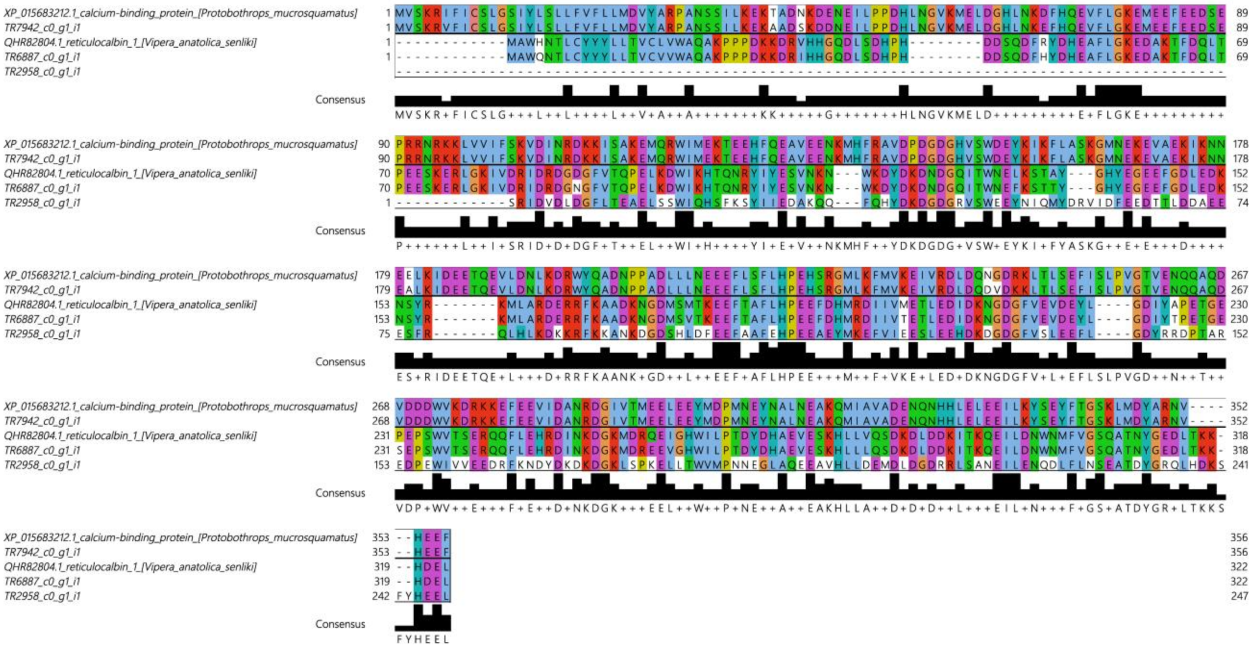

Figure S16. Multiple sequence alignment of **calcium-binding protein** (Reticulocalbin) transcripts in comparison to sequences of representative venomous snakes. Alignment generated by MUSCLE with ClustalX Colour Scheme.

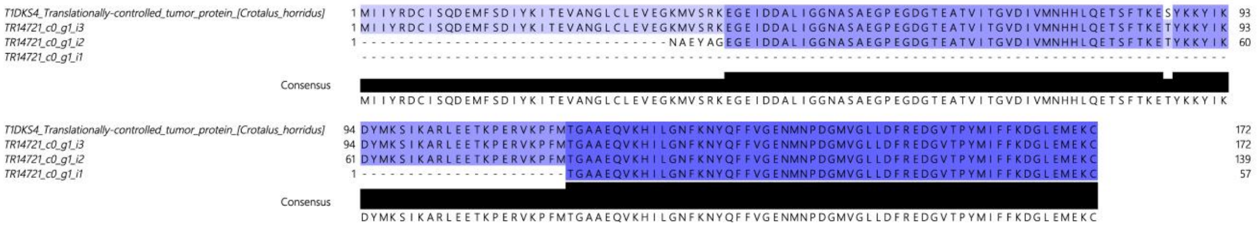

Figure S17. Multiple sequence alignment of **translationally-controlled tumor protein** (TCTP/HRF) transcripts from the venom gland transcriptome of *A. feae* in comparison with *Crotalus horridus* homolog. Alignment generated by MUSCLE with percentage Identity coloring scheme.

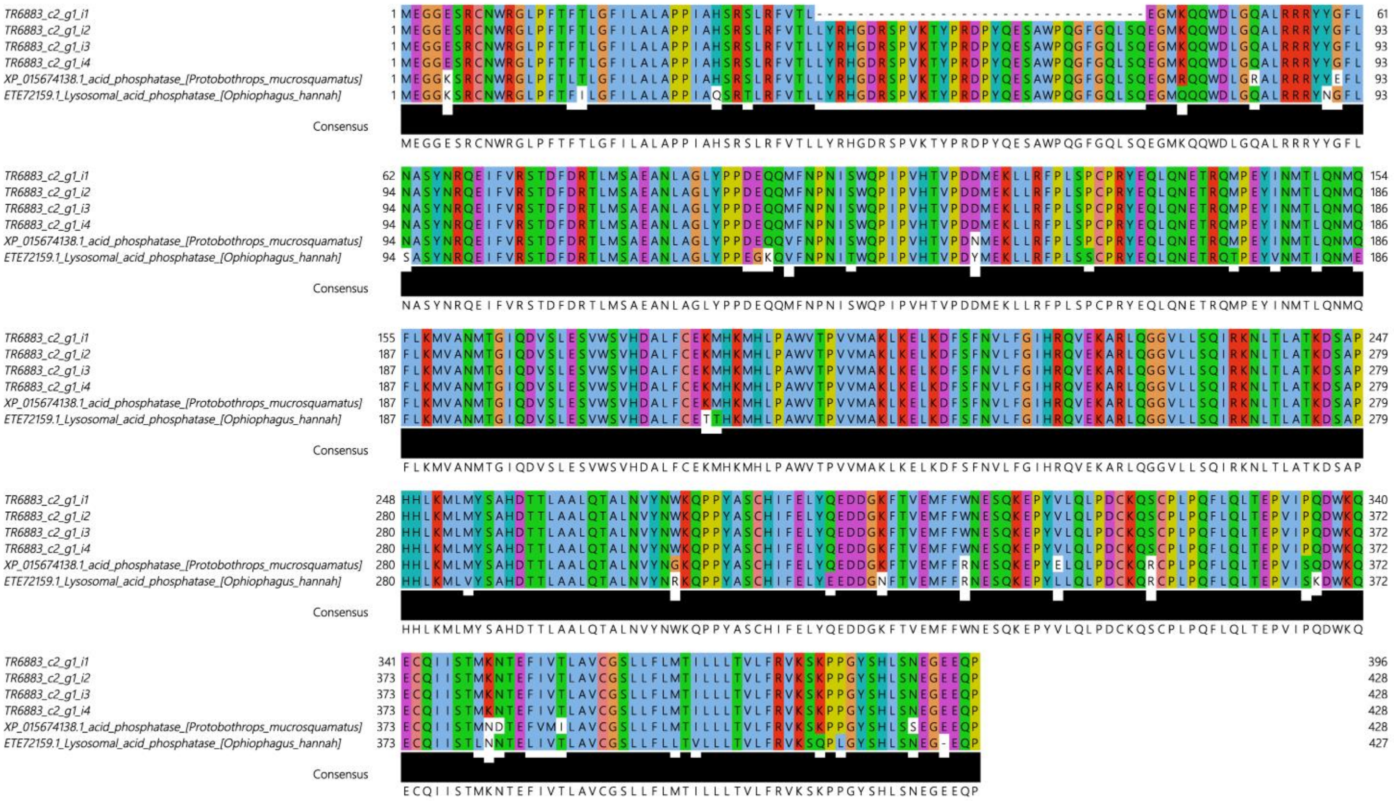

Figure S18. Multiple sequence alignment of **lysosomal acid phosphatase** (vAP) from the venom gland transcriptome of *A. feae* in comparison to vAP sequences of representative venomous snakes. Alignment generated by MUSCLE with percentage Identity coloring scheme.

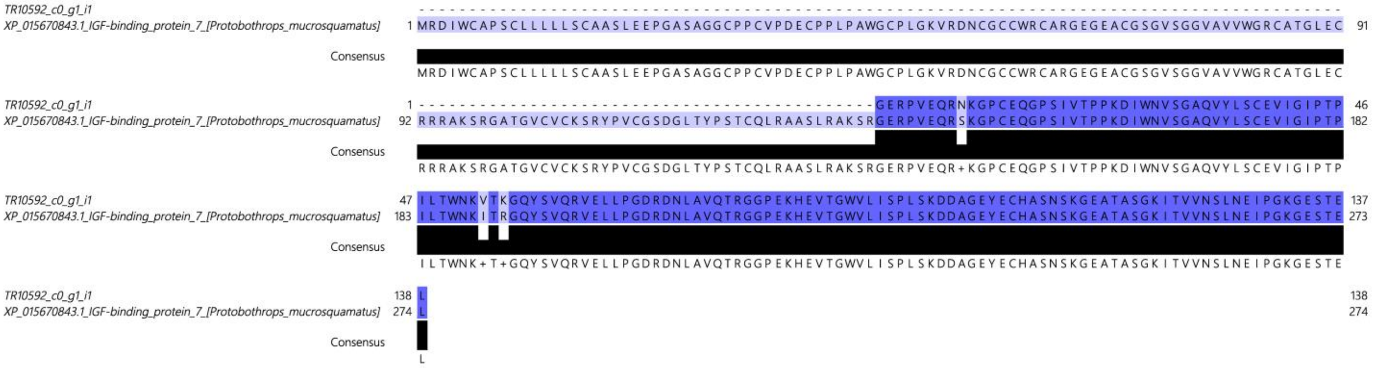

Figure S19. Sequence alignment of **Insulin-like growth factor-binding protein** (IGFBP) partial transcript from the venom gland transcriptome of *A. feae* in comparison with *Protobothrops mucrosquamatus* homolog. Alignment generated by MUSCLE with percentage Identity coloring scheme.

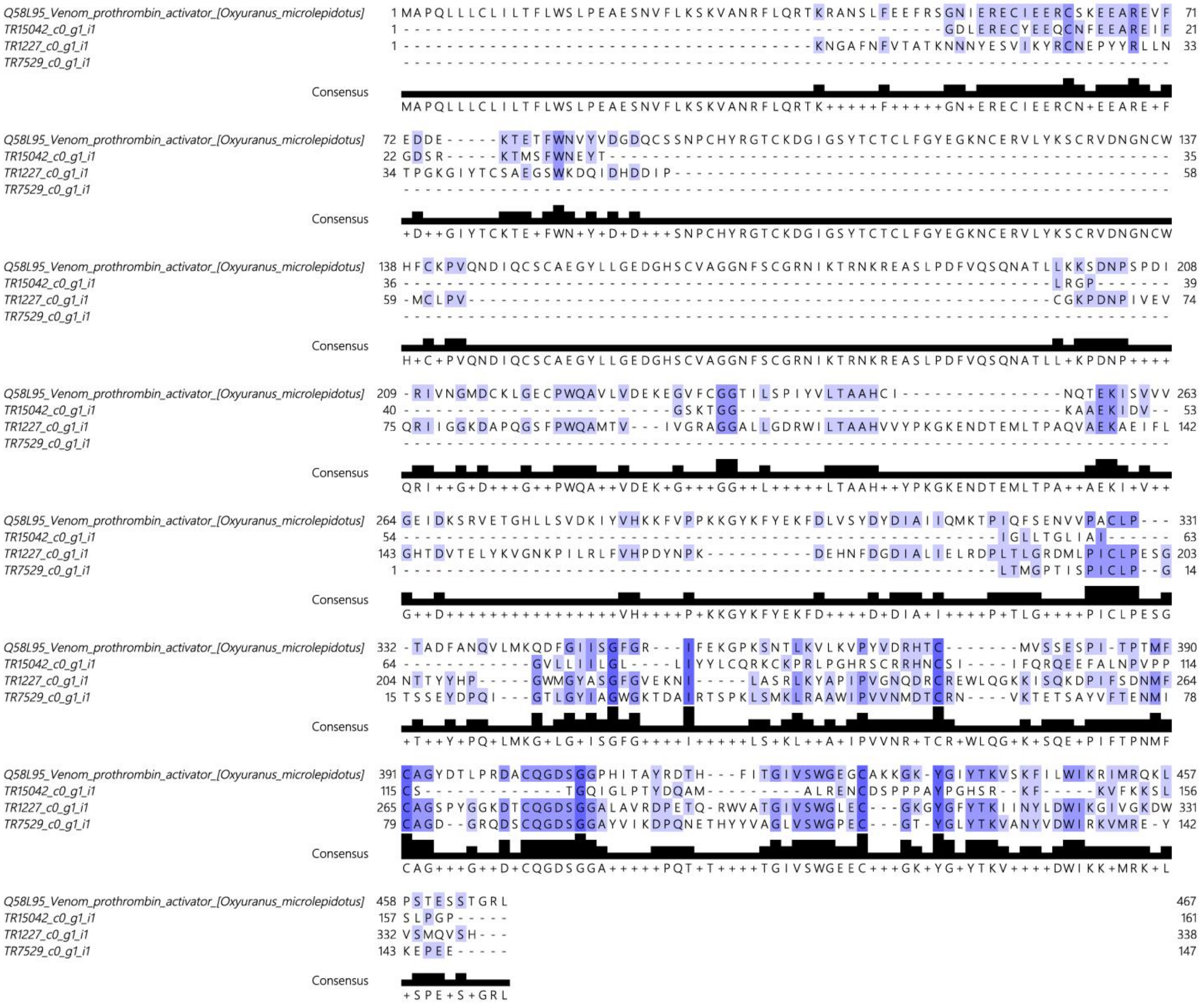

Figure S20. Multiple sequence alignment of **venom prothrombin activator** (vPA) partial transcripts from the venom gland transcriptome of *A. feae* in comparison with *Oxyuranus microlepidotus* homolog. Alignment generated by MUSCLE with percentage Identity coloring scheme.

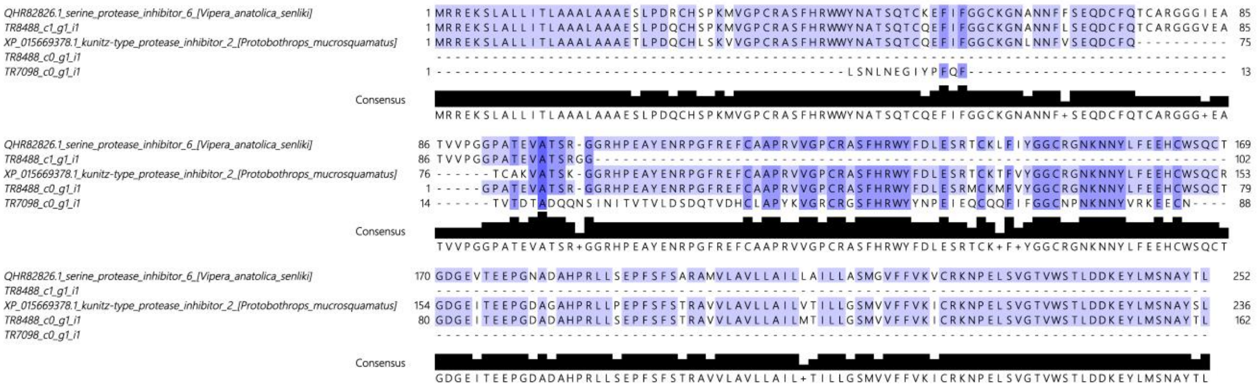

Figure S21. Multiple sequence alignment of **kunitz** (KUN) domains with comparison to kunitz-like sequences of representative venomous snakes. Alignment generated by MUSCLE with percentage Identity coloring scheme.

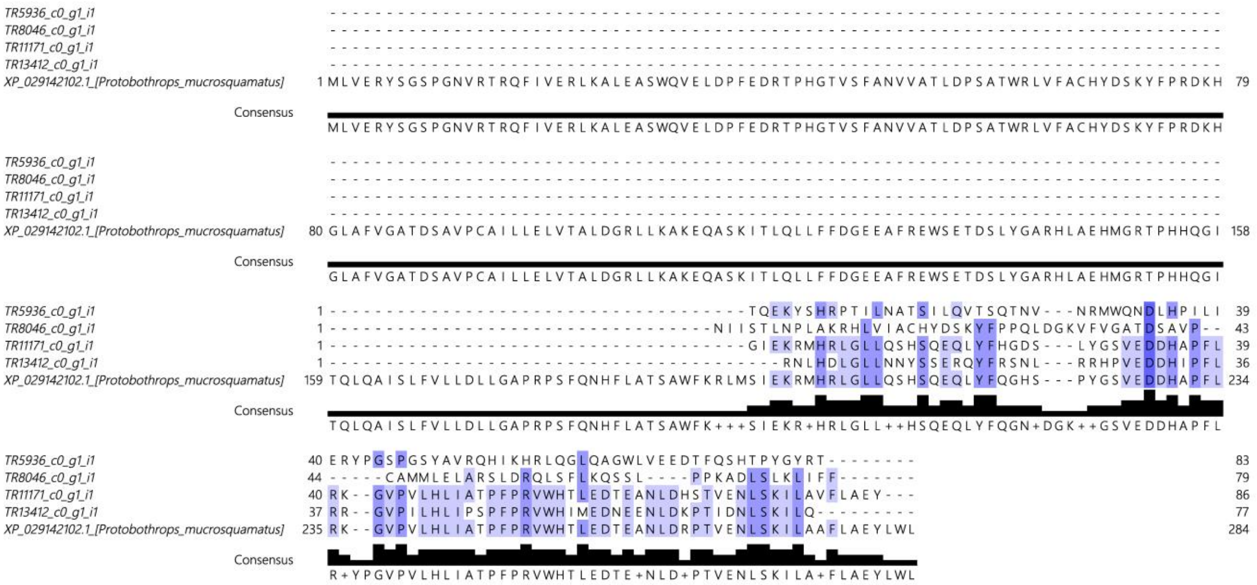

Figure S22. Multiple sequence alignment of **glutaminyl-peptide cyclotransferase** (QC) partial transcripts domains with *Protobothrops mucrosquamatus* homolog. Alignment generated by MUSCLE with percentage Identity coloring scheme.

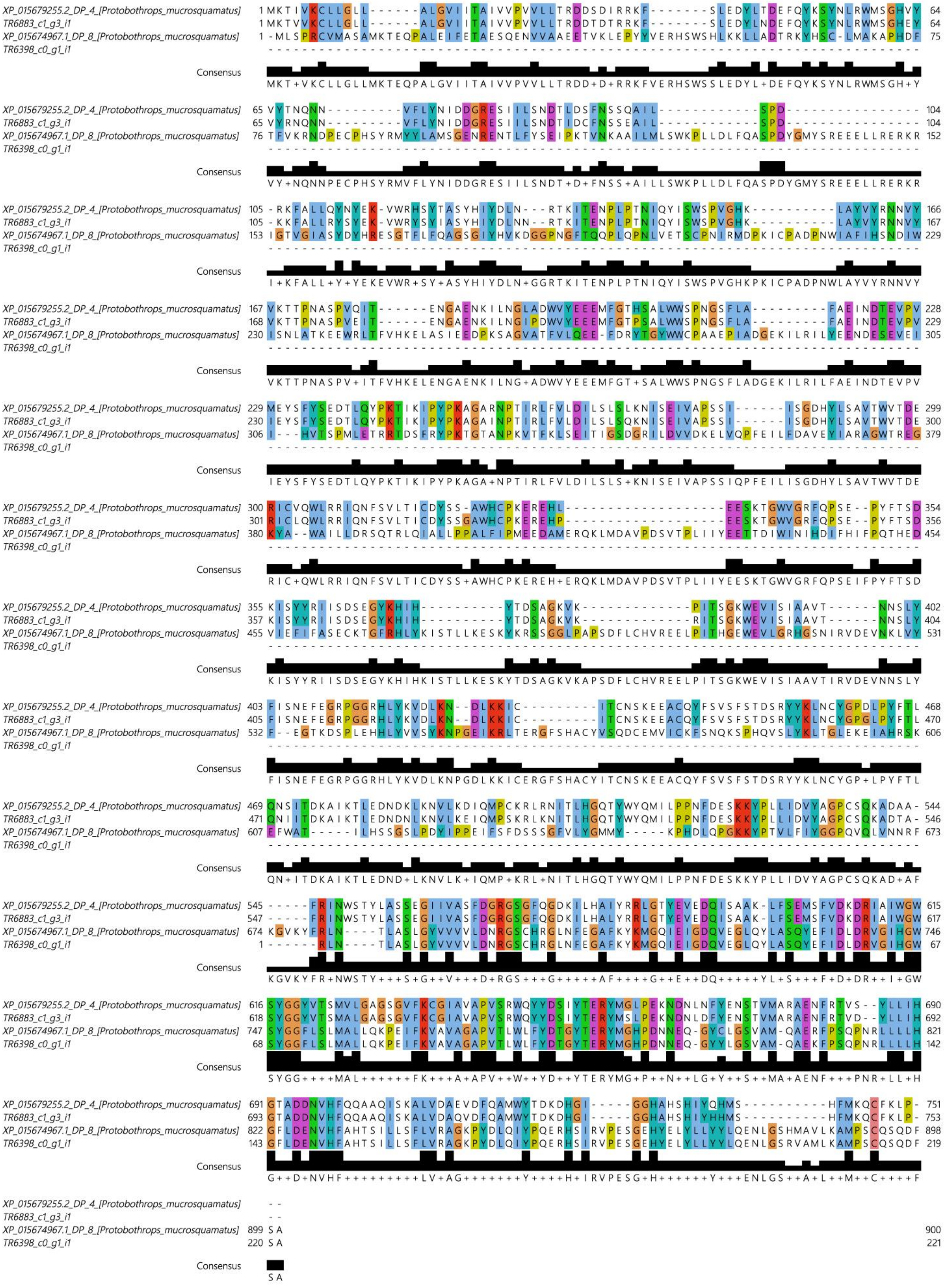

Figure S23. Multiple sequence alignment of **dipeptidyl peptidase** (vDP) transcripts in comparison with *Protobothrops mucrosquamatus* homologs. Alignment generated by MUSCLE with ClustalX Colour Scheme.

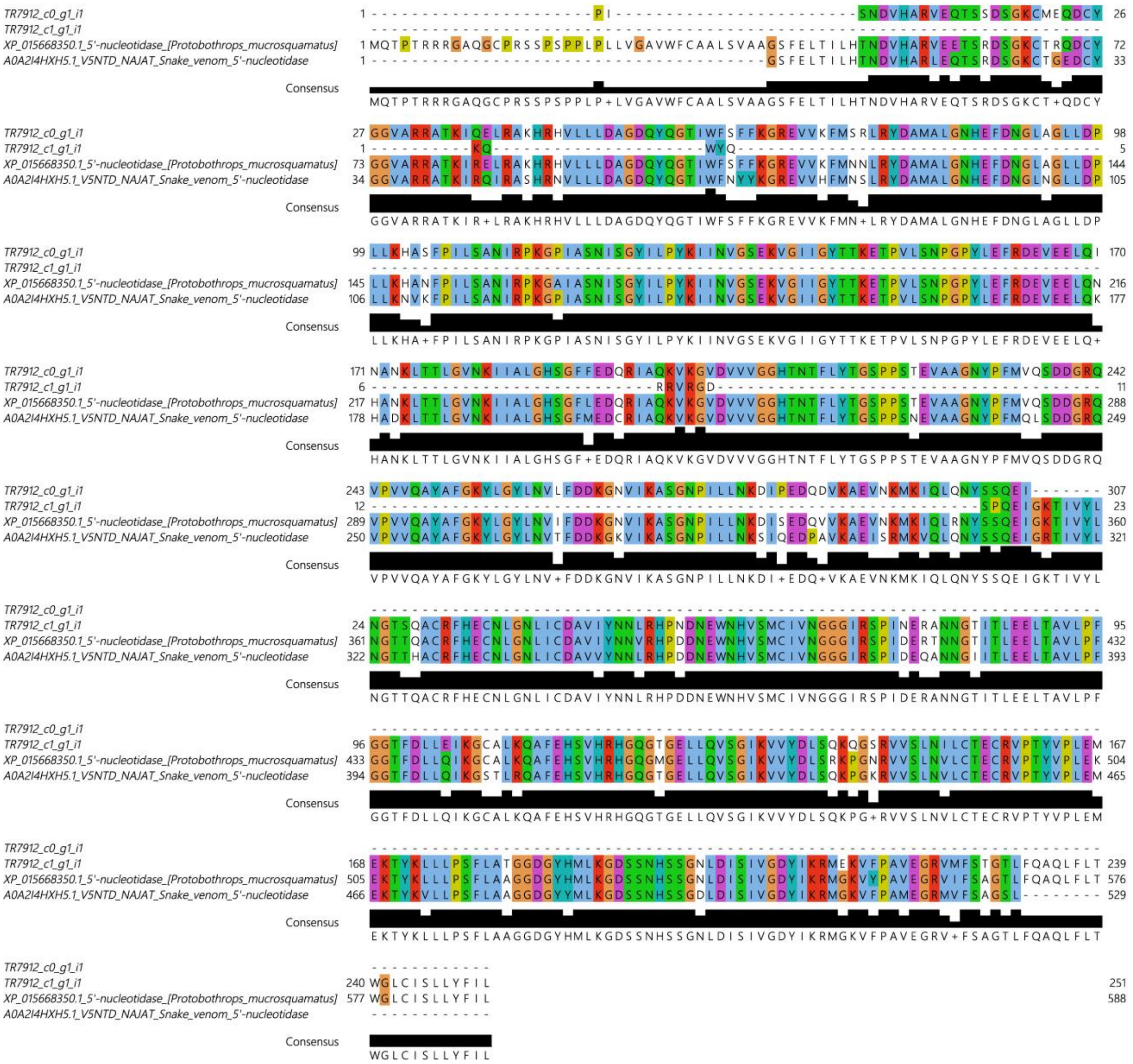

Figure S24. Multiple sequence alignment of **5'-nucleotidase** (NUC) from the venom gland transcriptome of *A. feae* in comparison to **5'-nucleotidase** sequences of representative venomous snakes. Alignment generated by MUSCLE with ClustalX Colour Scheme.

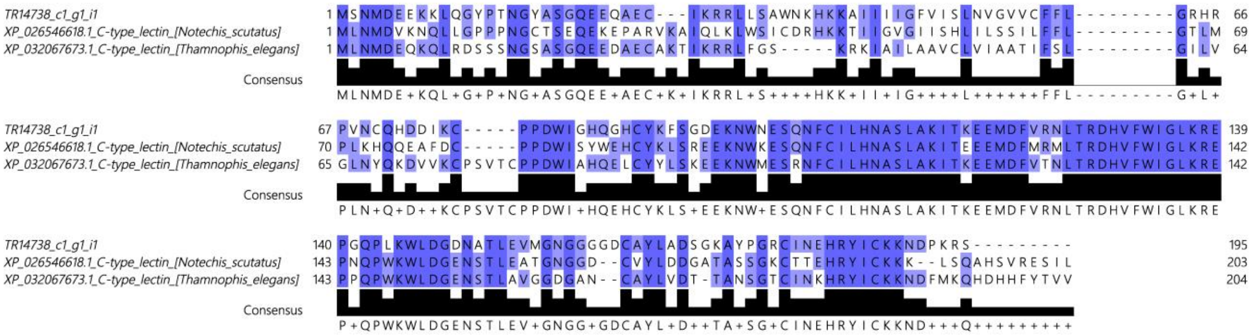

Figure S25. Multiple sequence alignment of **C-type lectins** with other snaclecs. Alignment generated by MUSCLE with percentage Identity coloring scheme.

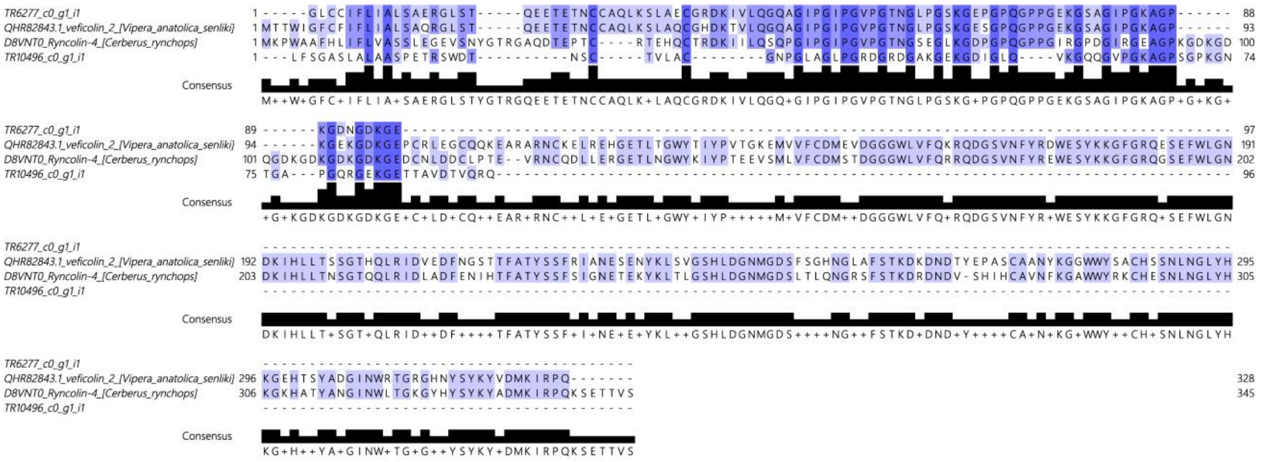

Figure S26. Multiple sequence alignment of **fibrinogen-like** partial transcripts from the venom gland transcriptome of *A. feae* in comparison to fibrinogen-like sequences of representative venomous snakes. Alignment generated by MUSCLE with percentage Identity coloring scheme.

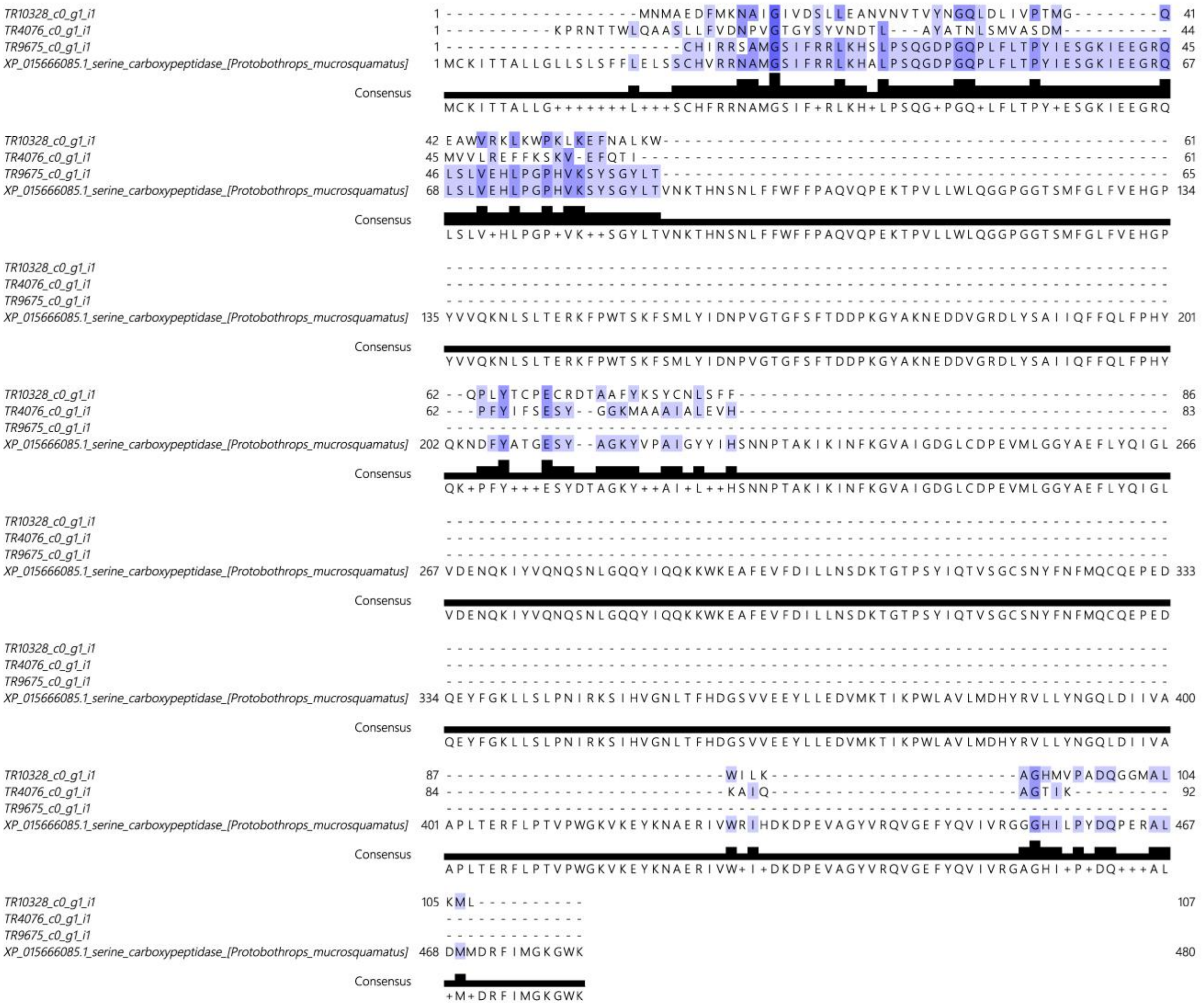

Figure S27. Multiple sequence alignment **serine carboxypeptidase** (vSC) partial transcripts with *Protobothrops mucrosquamatus* homolog. Alignment generated by MUSCLE with percentage Identity coloring scheme.

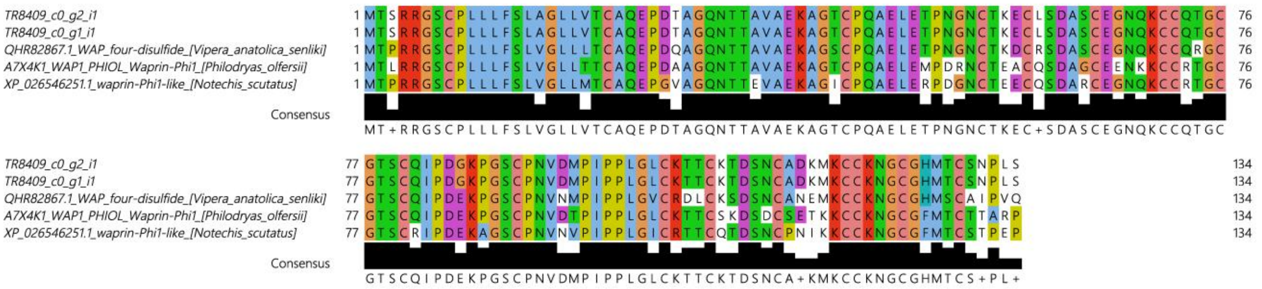

Figure S28. Multiple sequence alignment of **waprin** transcripts in comparison to waprin sequences of representative venomous snakes. Alignment generated by MUSCLE with ClustalX Colour Scheme.

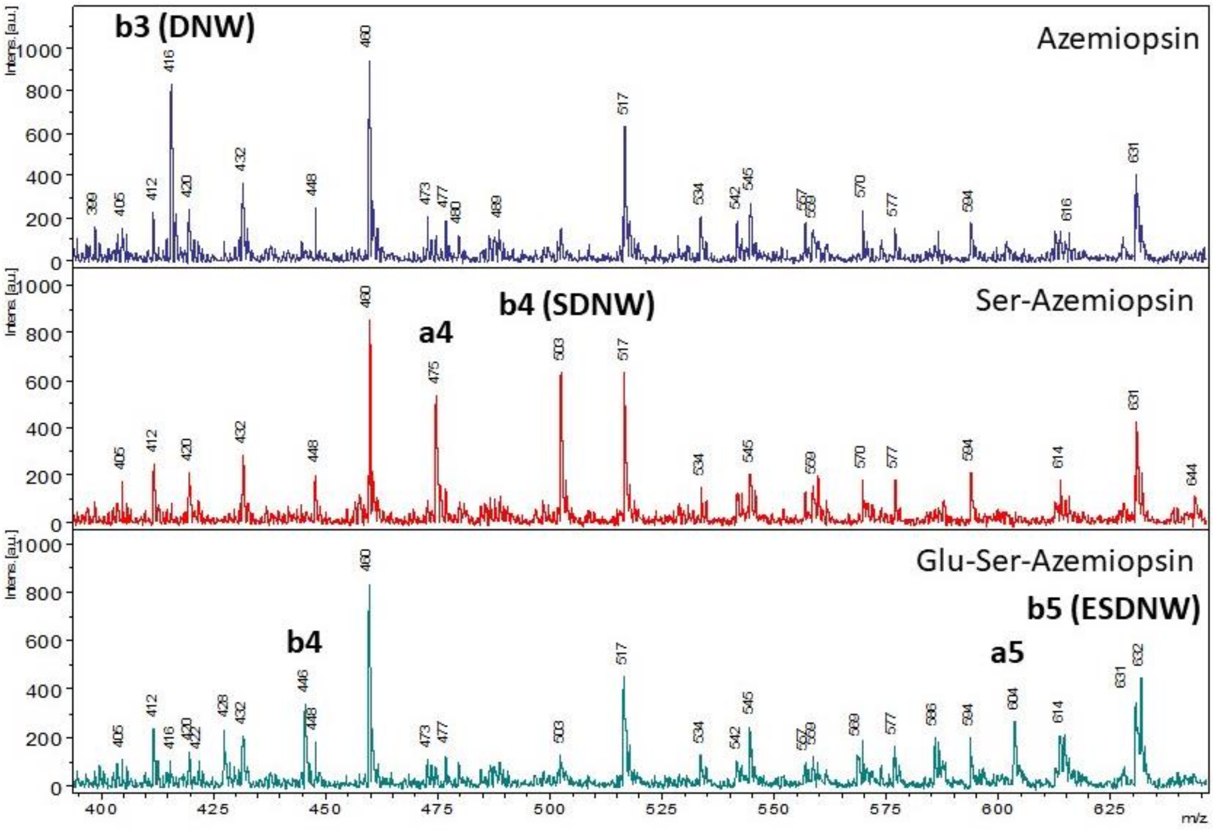

Figure S29. Sections of MS/MS spectra of azemiopsin and ist N-terminally extended analogues; shift of the b-ions (azemiopsin 416 = b3, DNW; Ser-azemiopsin 503 = b4, SDNW; Glu-Ser-Azemiopsin, 632 = b5, ESDNW).

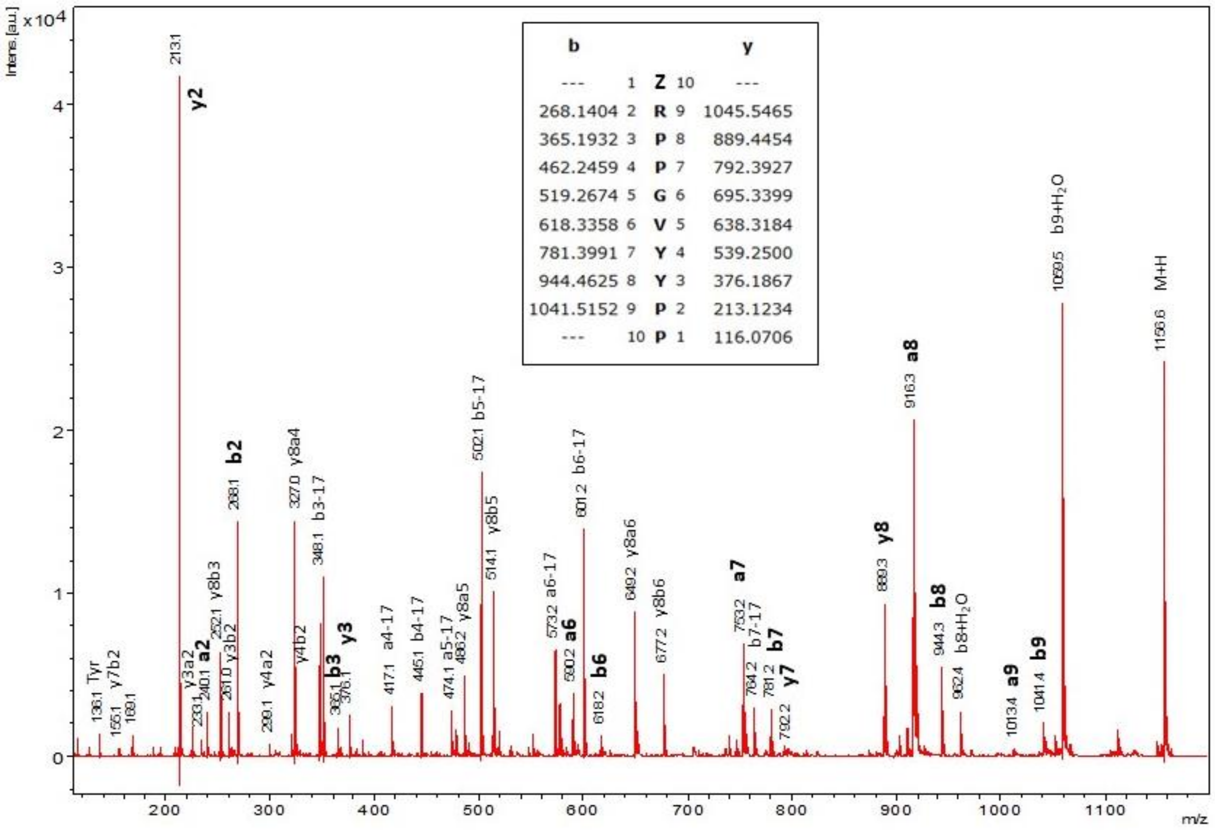

Figure S30. MS/MS spectrum of R-BPP and its interpretation; the insert shows the theoretical b- and y-ions for the sequence ZRPPGVYYPP, with Z for N-terminal pyroglutamic acid (theoretical mass 1156.579); strong internal ions are observed due to preferential fragmentation at Pro.
